# Transcriptional regulation and chromatin architecture maintenance are decoupled functions at the *Sox2* locus

**DOI:** 10.1101/gad.349489.122

**Published:** 2022-06-01

**Authors:** Tiegh Taylor, Natalia Sikorska, Virlana M. Shchuka, Sanjay Chahar, Chenfan Ji, Neil N. Macpherson, Sakthi D. Moorthy, Marit A.C. de Kort, Shanelle Mullany, Nawrah Khader, Zoe E. Gillespie, Lida Langroudi, Ian C. Tobias, Tineke L. Lenstra, Jennifer A. Mitchell, Tom Sexton

**Affiliations:** 1 Department of Cell and Systems Biology, University of Toronto, Toronto, Ontario M55 3G5, Canada;; 2 Institute of Genetics and Molecular and Cellular Biology (IGBMC), UMR7104, Centre National de la Recherche Scientifique, U1258, Institut National de la Santé et de la Recherche Médicale, University of Strasbourg, 6704 Illkirch, France;; 3 Division of Gene Regulation, the Netherlands Cancer Institute, Oncode Institute, 1066CX Amsterdam, the Netherlands

**Keywords:** enhancer, transcription, chromatin loop, TAD, allele-specific, genome engineering, CTCF

## Abstract

How distal regulatory elements control gene transcription and chromatin topology is not clearly defined, yet these processes are closely linked in lineage specification during development. Through allele-specific genome editing and chromatin interaction analyses of the *Sox2* locus in mouse embryonic stem cells, we found a striking disconnection between transcriptional control and chromatin architecture. We traced nearly all *Sox2* transcriptional activation to a small number of key transcription factor binding sites, whose deletions have no effect on promoter–enhancer interaction frequencies or topological domain organization. Local chromatin architecture maintenance, including at the topologically associating domain (TAD) boundary downstream from the *Sox2* enhancer, is widely distributed over multiple transcription factor-bound regions and maintained in a CTCF-independent manner. Furthermore, partial disruption of promoter–enhancer interactions by ectopic chromatin loop formation has no effect on *Sox2* transcription. These findings indicate that many transcription factors are involved in modulating chromatin architecture independently of CTCF.

## Abstract (precis)

Here, Taylor et al. investigated how distal regulatory elements control gene transcription and chromatin topology in lineage specification during development. Through allele-specific genome editing and chromatin interaction analyses of the Sox2 locus in mouse embryonic stem cells, they found a striking disconnection between transcriptional control and chromatin architecture, and traced nearly all Sox2 transcriptional activation to a small number of key transcription factor binding sites, whose deletions have no effect on promoter–enhancer interaction frequencies or topological domain organization.

Enhancer sequences are critical positive regulators of gene transcription that ensure appropriate spatiotemporal control of gene expression during development and in adult tissues (Grosveld et al. 2021). Enhancers can regulate single or multiple genes (Andersson et al. 2014; Fukaya et al. 2016; Moorthy et al. 2017; Allahyar et al. 2018), and skip over adjacent genes to modulate specific targets from megabase-level distances (Lettice et al. 2003; Sanyal et al. 2012). Across different tissues, chromatin modifications are more dynamic at enhancers than at promoters (Roadmap Epigenomics Consortium 2015), and many genes are regulated by different enhancers in various cellular contexts (Rodríguez-Carballo et al. 2017; Bahr et al. 2018; Maqbool et al. 2020), suggesting that most epigenetic information is encoded at enhancers. How enhancers are able to activate transcription and how they regulate the appropriate gene or genes without activating nontarget genes within the same chromatin region remain open questions.

Enhancers and regulated genes are often spatially configured by chromatin–chromatin interactions, whereby gene enhancer groups, which are linearly distant on the 2D chromosome fiber, may be brought into close proximity in 3D nuclear space (Carter et al. 2002; Tolhuis et al. 2002; Palstra et al. 2003; Sanyal et al. 2012; Schoenfelder et al. 2015). Growing evidence suggests that long-range chromatin interactions are mediated by loop extrusion wherein the ring-like cohesin complex translocates bidirectionally along chromatin, bringing linearly distal regions near to one another (Sanborn et al. 2015; Fudenberg et al. 2016; Davidson et al. 2019; Li et al. 2020b). Chromosome conformation capture approaches have shown that the genome is partitioned into topologically associating domains (TADs) that can insulate genes in adjacent TADs from enhancer activity outside their TAD (Dixon et al. 2012; Sexton et al. 2012; Lupiàñez et al. 2015). The interaction of CCCTC-binding factor (CTCF) with the genome is enriched at TAD boundaries, and the orientation of asymmetric CTCF motifs within these boundaries has a role in maintaining TAD structures (de Wit et al. 2015; Guo et al. 2015; Sanborn et al. 2015; Nora et al. 2017). In other contexts, CTCF has been associated with insulator function, binding and stabilizing cohesin (Bell et al. 1999; Wendt et al. 2008; Phillips and Corces 2009), or a means of anchoring distal enhancers to promoter-proximal CTCF-bound sites (Schuijers et al. 2018; Kubo et al. 2021). The extent to which CTCF-associated regions are generally required components for chromatin–chromatin contact maintenance, however, is debatable, since removal of such sites at other selected genomic loci has only negligible effects on chromatin topology and gene expression profiles (de Wit et al. 2015; Despang et al. 2019). Alternatively, long-range chromatin interactions can be mediated by transcription factors bound to DNA, potentially via protein dimerization events (Deng et al. 2012), clustering into nuclear hubs (Tolhuis et al. 2002; Mitchell and Fraser 2008; Schoenfelder et al. 2010; Li et al. 2020a), and/or formation of phase-separated condensates (Chong et al. 2018; Wei et al. 2020). Transcription factors are also able to anchor cohesin and therefore may modulate loop extrusion events (Liu et al. 2021; Vos et al. 2021). Despite a clear requirement for cohesin loading/unloading dynamics in maintaining genomic architecture, perturbation studies reveal conflicting and often weak corresponding effects on the transcriptome (Rao et al. 2017; Schwarzer et al. 2017; Liu et al. 2021). Additionally, depletion of proteins involved in condensate formation at enhancers has been shown to disrupt transcription but not long-range interactions (Crump et al. 2021), demonstrating that the relationship between enhancer–promoter interactions and transcription is still not well understood.

The *Sox2* (sex-determining region Y-box 2) gene encodes a transcription factor necessary for pluripotency and self-renewal in mouse embryonic stem cells (ESCs) and embryonic development (Avilion et al. 2003; Thomson et al. 2011). Deletion analyses revealed that *Sox2* transcription in mouse ESCs and the developing epiblast is regulated by the *Sox2* control region (SCR), a 7.3-kb cluster of transcription factor-bound regions located >100 kb downstream from *Sox2* (Chen et al. 2012; Li et al. 2014; Zhou et al. 2014; Chakraborty et al. 2022). The *Sox2* gene and the SCR are each at the border of an ESC-specific TAD that is lost upon differentiation to SOX2-dependent neural precursor cells (Bonev et al. 2017) and absent in other cell types not expressing *Sox2* (Hu et al. 2018; Stadhouders et al. 2018). Additionally, *Sox2* and the SCR appear to interact in ESCs through the formation of a chromatin loop that excludes most of the intervening DNA (Zhou et al. 2014; de Wit et al. 2015; Ben Zouari et al. 2020; Huang et al. 2021). A larger 27-kb region, comprising the SCR and two additional transcription factor-bound regions, was previously identified as a “superenhancer” (Whyte et al. 2013), a class of genomic element originally proposed to contain multiple synergistic activators of target gene transcription. More recently, genomic interrogations of enhancer clusters have questioned the “superenhancer” theory given that individual regions within these clusters have largely redundant functions (Hay et al. 2016; Moorthy et al. 2017). These findings are consistent with the concept of shadow enhancers, or regulatory elements that confer phenotypic robustness through their partially redundant activities (Perry et al. 2010). At the *Sox2* locus, it remains unclear how these multiple transcription factor-bound subunits within and surrounding the SCR contribute to *Sox2* transcription control or locus topology in ESCs.

To identify the sequences required for *Sox2* transcription as well as those involved in ESC-specific chromatin topology, we used allele-specific CRISPR/Cas9-mediated deletions to systematically remove all transcription factor-bound regions within and surrounding the SCR “superenhancer.” We found that two such regions within the SCR, *Sox2* regulatory regions (SRRs) 107 and 111 (numerically designated according to their distance from the *Sox2* promoter), are responsible for the majority of *Sox2* transcription in ESCs; however, the deletion of these regions had no effect on interaction frequency between the SCR and the *Sox2* gene. These data show a stark uncoupling of transcription enhancement from chromatin–chromatin interaction maintenance. Furthermore, removal of the sole CTCF-bound site within the SCR had no effect on either chromatin topology or *Sox2* transcription. Significant perturbation of chromatin interaction frequencies and TAD border insulation function required deletion of the entire SCR, comprising multiple transcription factor-bound sites beyond those responsible for gene activation. On the other hand, insertion of CTCF motifs between *Sox2* and the SCR was able to partially insulate these sites from each other from a topological standpoint, but with no effect on transcription. Thus, whereas enhancer function is mediated by a small number of key transcription factor-bound regions, chromatin–chromatin interaction is independent from transcriptional control and maintained in a distributed manner by many elements within the *Sox2* TAD.

## Results

### Deletion of the SCR partially disrupts chromatin interactions with the *Sox2* gene in ESCs

**Figure 1.**
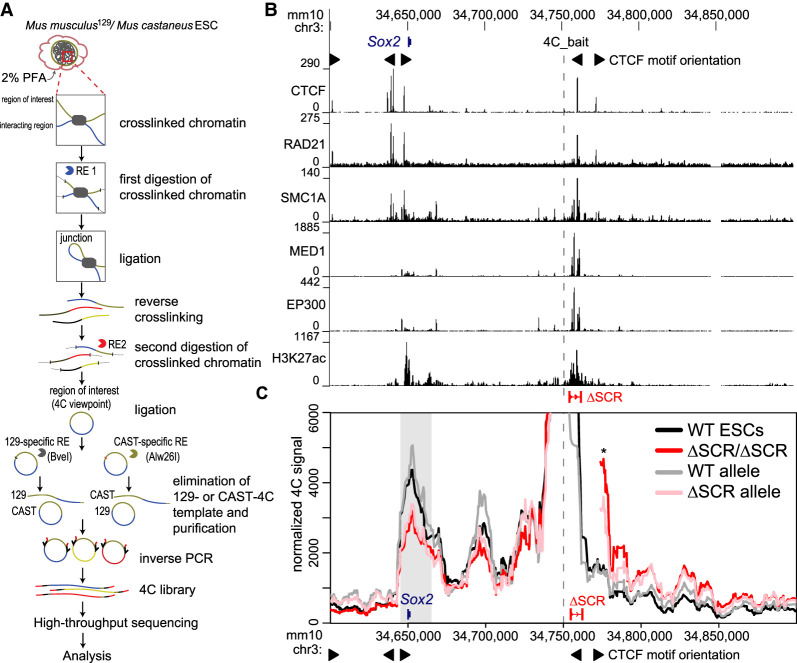
Deletion of the SCR partially disturbs chromatin interactions with the *Sox2* gene in ESCs. (*A*) Schematic of the allele-specific 4C approach. (RE) Restriction enzyme, (PFA) paraformaldehyde. (*B*) The region surrounding the *Sox2* gene is displayed on the UCSC genome browser (mm10). The SCR deletion (ΔSCR) is shown, and the 4C bait region is indicated as a dashed line. ChIP-seq conducted in ESCs is shown *below* for CTCF, RAD21, SMC1A, MED1, EP300, and H3K27ac. The motif orientations of bound CTCF sites are denoted. (*C*) 4C data are shown for wild-type cells (WT, black, *n* = 4), homozygous ΔSCR/ΔSCR cells (red, *n* = 4), and heterozygous ΔSCR cells. Data from the heterozygous cells are displayed separately for the WT (gray, *n* = 3) and ΔSCR (pink, *n* = 4) alleles. The dashed line indicates the location of the 4C bait region. The gray box indicates the bait-interacting region surrounding the *Sox2* gene. Compared with WT cells, a significant decrease in relative interaction frequency of the 4C bait region with the *Sox2* gene was observed for homozygous ΔSCR/ΔSCR cells (*P* = 0.02) and the ΔSCR allele in heterozygous ΔSCR cells (*P* = 0.04), but not the WT allele in heterozygous ΔSCR cells (*P* = 0.46). An asterisk denotes the region now contiguous with the bait in ΔSCR alleles, explaining the very high 4C signal. For deletion alleles, the 4C signal has been omitted from the deleted region and flanking positions that are also affected by the deletion when computing running means. The motif orientations of bound CTCF sites are denoted.

Deletion of the SCR abrogates the majority of *Sox2* transcription in ESCs (Li et al. 2014; Zhou et al. 2014); however, the effect of SCR removal on the conformation of the locus had not been investigated. We examined the relationship between the loss of the SCR and the ESC-specific chromatin architecture profile at the *Sox2* locus. To establish a locus-wide view of the chromatin contacts in both wild-type F1 ESCs (*Mus musculus*^129^ × *Mus castaneus*) and ESCs containing a homozygous deletion of the SCR (ΔSCR/ΔSCR), we subjected fixed chromatin from both lines to an allele-specific 4C-seq approach (adapted from Splinter et al. 2011) using a bait region located just upstream of the SCR (Fig. 1A,B). Aside from the *Sox2* promoter, the SCR displays the most enrichment of H3K27ac and binding of EP300, MED1, SMC1A, RAD21, and CTCF within the locus (Fig. 1B). Regions interacting with the bait at appreciably higher levels than expected from a fitted background model (Geeven et al. 2018) were called for each biological replicate, and an interaction between the SCR-proximal bait and *Sox2* gene was reproducibly identified (Fig. 1C; Supplemental Table S1; Supplemental Fig. S1A). The apparent large increase in interaction just downstream from the SCR in the deletion line (Fig. 1C, asterisk) is a direct result of this downstream region now being directly contiguous to the 4C bait. Notably, we did observe that SCR deletion caused increased interaction with regions further downstream from the SCR, which is discussed further below. 4C from the *Sox2* promoter bait also showed a strong interaction with the SCR in wild-type ESCs, with expected lack of interaction at both the deleted SCR and intact sequence just downstream from the SCR (Supplemental Fig. S1B). The *Sox2*-spanning region, which forms an interaction with the SCR-proximal region in all wild-type 4C replicates, was used for quantitative interaction comparisons with all other tested clonal cell lines. Relative to wild-type cells, ΔSCR/ΔSCR cells showed a significant 28% decrease (*P* = 0.02) in relative contact frequency between the SCR-proximal bait and *Sox2* (Fig. 1C). This finding suggested that regions within the SCR contribute to the maintenance of ESC-specific genomic configurations at the *Sox2* locus. An alternative mechanistic explanation, however, was also possible: ESCs containing a homozygous deletion of the SCR are partially differentiated and exhibit markedly reduced SOX2 protein levels (Zhou et al. 2014). We therefore raised the question of whether the observed reduction in SCR–*Sox2* gene interaction frequency in these cells was mediated by *trans* mechanisms associated with the depletion of SOX2 protein, which might be required to anchor these chromatin contacts, rather than by deletion of the *Sox2* enhancer DNA in *cis*. We assessed allele-specific contact frequencies across the *Sox2* locus in cells carrying a heterozygous deletion of the SCR, which contained wild-type-equivalent SOX2 protein levels (Zhou et al. 2014). The allele containing an intact SCR (Fig. 1C, WT allele) exhibited a chromatin contact profile that mirrored that observed in wild-type cells (*P* = 0.46), whereas the relative *Sox2*–SCR contact frequency of the allele with the deletion was reduced by 24% (ΔSCR allele; *P* = 0.04) compared with wild-type levels. This loss is not significantly different from the reduction observed in cells containing homozygous deletion of the SCR (*P* = 0.74) (Fig. 1C). When comparing homozygous and heterozygous counterparts of the wild-type or deleted alleles, no other region demonstrated reproducible differences in interaction strength (Supplemental Fig. S1A). These results thus indicate that the loss of the SCR in *cis* directly accounts for reduced interactions with the *Sox2* gene. Interestingly, the majority of the interaction is apparently maintained with complete loss of the SCR, which confers >80% of *Sox2* transcriptional activity. These data suggest that genome architecture and expression control may be decoupled at this locus, and that additional *cis* elements are required to maintain local chromatin structure.

### Two transcription factor-bound regions are jointly responsible for SCR-mediated enhancement of *Sox2* transcription

**Figure 2.**
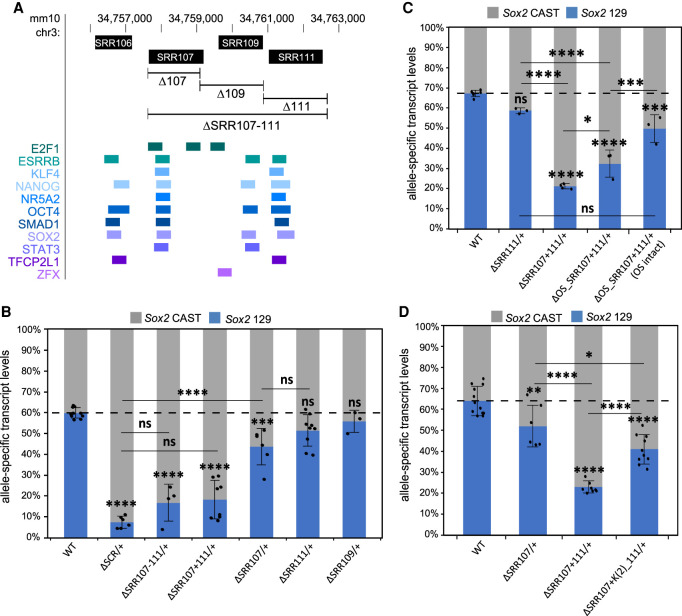
SRR107 and SRR111 have a partially redundant role and are required for the transcription-enhancing capacity of the SCR. (*A*) The SCR genomic region is displayed on the UCSC genome browser (mm10). *Sox2* regulatory regions (SRRs, *top*) correspond to transcription factor-bound regions derived from ESC ChIP-seq data sets compiled in the CODEX database (*bottom*). In *B–D*, allele-specific primers detect *Sox2 musculus* (129; blue) or *castaneus* (CAST; gray) mRNA by RT-qPCR from F1 ESC clones from the indicated genotype. Expression levels for each allele are shown relative to the total transcript levels. Error bars represent SD. *n* ≥ 3. Significant differences from the WT values are indicated. (*) *P* < 0.05, (**) *P* < 0.01, (***) *P* < 0.001, (****) *P* < 0.0001, (ns) not significant. (*B*) Deletion of both SRR107 and SRR111 (ΔSRR107–111/+; ΔSRR107+111/+) causes a reduction in *Sox2* transcript levels similar to that for deletion of the entire SCR. (*C*) Deletion of the OCT4:SOX2 (OS) motif in SRR107 (ΔOS_SRR107+111/+) reduces *Sox2* transcript levels on the linked allele. Clones with nucleotide deletions near but not disrupting the OS motif (ΔOS_SRR107+111/+ [OS intact]) displayed increased transcription of *Sox2* on the linked allele compared with clones with a deleted OS motif. (*D*) Deletion of two KLF4 motifs in SRR111 [ΔSRR107+K(2)_111/+] reduces *Sox2* transcript levels on the linked allele.

We next sought to assess which subregions within the SCR contribute to *Sox2* transcription. We previously established that, of the four transcription factor-bound regions within the SCR (SRR106, SRR107, SRR109, and SRR111) (Fig. 2A), only SRR107 and SRR111 are capable of up-regulating transcription of a reporter gene in ESCs (Zhou et al. 2014). To examine whether the same holds true in a genomic context, we created ESC clones with heterozygous deletions of either SCR subregion on the 129 allele (Supplemental Fig. S2; Supplemental Tables S2, S3). Allele-specific *Sox2* transcript-level quantification analysis (Moorthy and Mitchell 2016) allowed us to assess the endogenous activation potential of either region. We observed that the loss of SRR107 from the 129 allele is accompanied by a modest but significant alteration in the allelic ratio of *Sox2* transcripts, with a 27% reduction in transcript levels from the allele carrying the deletion (ΔSRR107/+) (Fig. 2B). Removal of SRR111 caused a weaker (14%), nonsignificant reduction in *Sox2* transcript production from the 129 allele (ΔSRR111/+) (Fig. 2B). A compound deletion made by deleting SRR111 on the 129 allele in a genomic background already lacking SRR107 on the same allele demonstrated a much larger (70%) decrease in allele-specific *Sox2* expression (ΔSRR107+111/+) (Fig. 2B). Furthermore, this reduction in transcript abundance was not statistically different from that observed in clones lacking the entire SCR, although we did note that the variation in expression levels was higher for these clones. Notably, the allelic imbalance of *Sox2* transcription was essentially the same whether or not the intervening region between SRR107 and SRR111 was also deleted, as indicated by the expression results for ΔSRR107–111/+ and ΔSRR107+111/+ cells (Fig. 2B). These data indicate that SRR107 and SRR111, acting in a partially redundant manner, underlie the majority of the transcriptional regulatory power of the SCR in coordinating ESC-specific *Sox2* transcription.

Since *Sox2* transcription was shown to be significantly reduced upon deletion of both SRR107 and SRR111 on the same allele, these enhancers were investigated for the presence of core ESC transcription factor binding motifs that may promote the activity of these regions. Using the JASPAR GeneReg database tool (Sandelin et al. 2004), we uncovered the presence of multiple high-scoring transcription factor motifs, including an OCT4:SOX2 composite motif in SRR107 and two KLF4 motifs in SRR111 (Supplemental Fig. S3A). Overlapping of ChIP-seq data sets extracted from the CODEX database (Sánchez-Castillo et al. 2015) confirmed the association of the corresponding transcription factor proteins over these sites (Fig. 2A; Supplemental Fig. S4). Because of the partially redundant functions of the two enhancers, we targeted each of these motifs for removal in clones carrying only one intact enhancer (in which the motif of interest resided) with the other SRR deleted on the same allele. Clones carrying microdeletions of the targeted motifs (Supplemental Fig. S3B) were subjected to allele-specific expression analyses. We found that deletion of the high-scoring OCT4:SOX2 motif in SRR107 (ΔOS_SRR107+111/+) resulted in a transcript reduction level close to that of the loss of the entire SRR107 in an SRR111 deletion-carrying background (Fig. 2C). Importantly, clones carrying slightly off-target microdeletions that retained an intact OCT4:SOX2 motif do not show as great a decrease in allele-specific *Sox2* transcript levels (ΔOS_SRR107+111/+ [OS intact]). Allele-specific ChIP analysis of OCT4:SOX2 motif-deleted cells revealed a disruption in the association of OCT4 and RNA polymerase II at the altered SRR (Supplemental Fig. S3C). Similar to previous heterozygous deletions of the SCR, heterozygous deletions of SCR subregions did not cause a reduction in SOX2 protein levels (Supplemental Fig. S3D; Zhou et al. 2014; Dhaliwal et al. 2019). The loss of both KLF4 motifs in SRR111 also caused a significant reduction of this region's activity in an SRR107 deletion-carrying background [ΔSRR107+K(2)_111/+] (Fig. 2D). These results suggest that these motifs, along with other motifs that have been shown to contribute to enhancer activity within the SCR (Singh et al. 2021), account for the reduced expression phenotype we observed upon deletion of the entire transcription factor-bound subregion. Collectively, these findings support a model for a distal gene regulation mechanism controlling *Sox2* transcription in ESCs that depends on ESC-specific transcription factors bound at these two regions.

### Enhancer activity and CTCF association are dispensable for distal chromatin contacts within the *Sox2* locus

**Figure 3.**
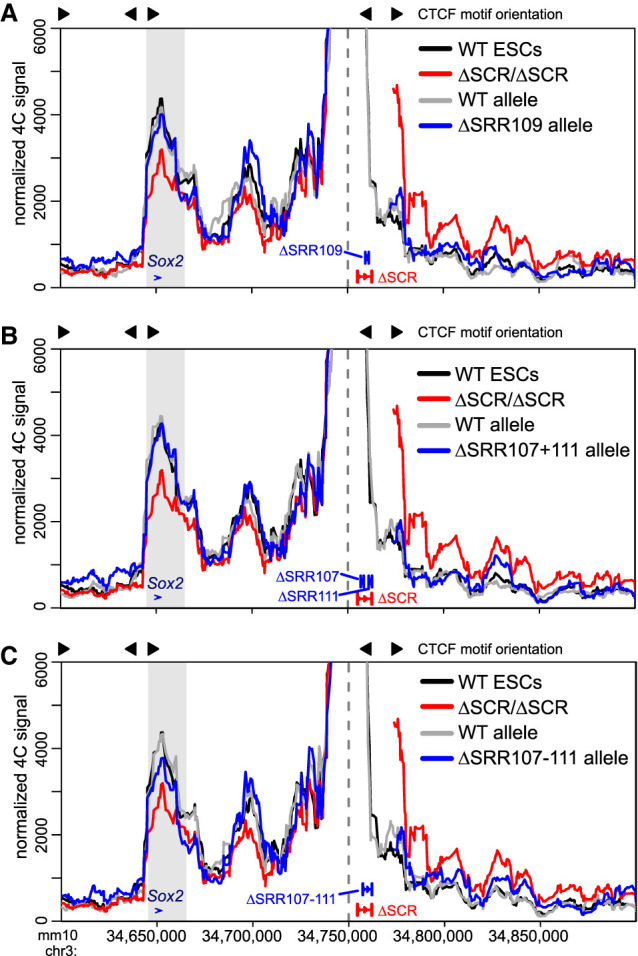
The SRR107 and SRR111 enhancers and the SRR109 CTCF-bound region are dispensable for the interaction between the SCR-proximal region and the *Sox2* gene. 4C data are shown for wild-type cells (WT, black, *n* = 4), homozygous ΔSCR/ΔSCR cells (red, *n* = 4), and heterozygous ΔSRR cells (gray/blue, *n* = 2 for each allele). Heterozygous deletions of SRR109 (*A*), SRR107 and SRR111 (*B*), or SRR107 to SRR111 (*C*) are shown in blue, with the WT allele shown in gray. In each figure, the dashed line indicates the location of the 4C bait region. The gray box indicates the bait-interacting region surrounding the *Sox2* gene, which is not significantly altered upon deletion of SRR109 (*P* = 0.4), SRR107 and SRR111 (*P* = 0.6), or SRR107 to SRR111 (*P* = 0.08). The motif orientations of CTCF-bound sites are shown *above* the plots. For deletion alleles, the 4C signal has been omitted from the deleted region and flanking positions that are also affected by the deletion when computing running means.

Aside from SRR107 and SRR111, which jointly drive the transcriptional enhancer activity for *Sox2* in ESCs, the SCR also contains a prominent CTCF binding site at SRR109 (Fig. 1B). CTCF has been associated with anchoring distal enhancers to promoter-proximal CTCF-bound sites (Kubo et al. 2021); however, a previous study identified only a slight decrease in the observed interaction frequency of the SCR with *Sox2* after biallelic removal of the core 16 bp within the SRR109 CTCF motif (de Wit et al. 2015). Additionally, acute CTCF depletion in ESCs had no effect on *Sox2* transcription (Nora et al. 2017). To assess whether SRR109 might function as an intra-SCR loop anchor, we generated a cell line containing a heterozygous deletion of SRR109 on the 129 allele, comprising the major CTCF site and some other transcription factor binding sites (Fig. 2; Supplemental Fig. S5), and subjected these clones to allele-specific 4C-seq and expression analysis. In line with previous perturbations of the SRR109 CTCF site (de Wit et al. 2015) and enhancer reporter assays (Zhou et al. 2014), heterozygous SRR109 deletion had only negligible effects on the allelic balance of *Sox2* expression levels (ΔSRR109/+) (Fig. 2B), indicating that this region has next to no direct enhancer activity in ESCs. Moreover, we observed no significant differences in 129 allele-derived chromatin–chromatin contact profiles between cells lacking one copy of SRR109 and wild-type cells (*P* = 0.4) (Fig. 3A), suggesting that this CTCF-bound element is not required for the genomic proximity between *Sox2* and the SCR. The observation that SRR107 and SRR111 are necessary for maintenance of *Sox2* transcription in ESCs led us to hypothesize that these two regions could be combinatorially responsible for anchoring chromatin interactions between the SCR-proximal region and the *Sox2* gene. Allele-specific 4C analysis of cells lacking both SRR107 and SRR111, however, revealed no significant differences in contact frequencies between the distal SCR-proximal region and *Sox2* in ΔSRR107+111/+ cells and wild-type cells (*P* = 0.6) (Fig. 3B). These findings support the notion that both SRR107 and SRR111 appear to be dispensable for the SCR–*Sox2* interaction.

We next considered that SRR109 might cooperate with the two enhancer components of the SCR to support its interaction with the *Sox2* promoter. To test this hypothesis, we used ΔSRR107–111/+ ESCs, which lacked both enhancers and the CTCF-bound region on the same allele (Supplemental Fig. S5). Here, we did observe a reduction (17%) in the chromatin interaction profile between the SCR-proximal region and the *Sox2* gene on the SRR107–SRR111 deletion-carrying allele compared with the corresponding wild-type allele. However, this observation did not meet the critical value for statistical significance in our analysis (*P* = 0.08) (Fig. 3C). This finding suggests that SRR107, SRR109, and SRR111 may minimally support the interaction of the SCR with the *Sox2* gene, although other genetic elements likely contribute to the bulk (∼83%) of the interaction. Overall, our results indicate a striking decoupling at this locus between genetic elements responsible for transcriptional activation (key transcription factor motifs within SRR107 and SRR111) and those influencing chromatin architecture, where the sole CTCF-bound site within the SCR appears to have only a minor contribution.

### A downstream CTCF-bound region is not responsible for the remaining *Sox2*–SCR-proximal region interaction in SCR deletion-carrying cells

**Figure 4.**
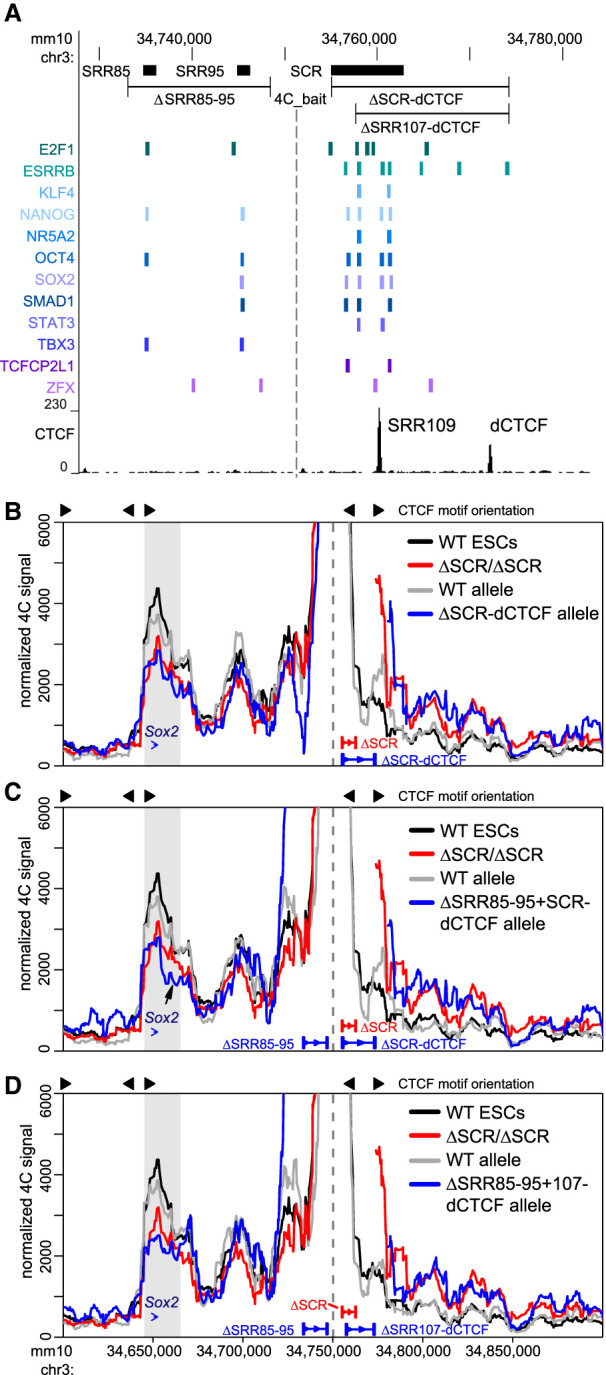
Additional CTCF- and transcription factor-bound regions surrounding the SCR support the interaction between the SCR-proximal region and the *Sox2* gene. (*A*) The transcription factor-bound regions surrounding the SCR are displayed on the UCSC genome browser (mm10). *Sox2* regulatory regions (SRRs) and the SCR (*top*) correspond to transcription factor-bound regions derived from ESC ChIP-seq data sets compiled in the CODEX database (*bottom*). (*Bottom*) CTCF ChIP-seq conducted in ESCs is shown, and the distal CTCF (dCTCF)-bound region is marked. In *B–D*, 4C data are shown for wild-type cells (WT, black, *n* = 4), homozygous ΔSCR/ΔSCR cells (red, *n* = 4), and heterozygous deletion-carrying cells (gray/blue, *n* = 2 for each allele). Heterozygous deletions of the SCR to dCTCF (*B*), SRR85 to SRR95 and SCR to dCTCF (*C*), or SRR85 to SRR95 and SRR107 to dCTCF (*D*) are shown in blue, with the WT allele shown in gray. In each panel, the dashed line indicates the location of the 4C bait region. The gray box indicates the bait-interacting region surrounding the *Sox2* gene. Compared with WT cells, a significant decrease in relative interaction frequency of the 4C bait region with the *Sox2* gene was observed for ΔSCR-dCTCF/+ (*P* = 0.006), ΔSRR85–95+SCR-dCTCF/+ (*P* = 0.005), or ΔSRR85–95+107-dCTCF/+ (*P* = 0.01) cells. In *C*, the arrow indicates a loss of interaction downstream from the *Sox2* gene after deletion of ΔSRR85–95+SCR-dCTCF/+. For deletion alleles, the 4C signal has been omitted from the deleted region and flanking positions that are also affected by the deletion when computing running means.

Since both homozygous and heterozygous SCR deletions were not sufficient to abolish chromatin interactions between the SCR-proximal region and the *Sox2* gene, we searched for other candidate regions surrounding the SCR that might support the bulk of the remaining interactions. We postulated that a CTCF-bound region (distal CTCF [dCTCF] in Fig. 4A; Supplemental Fig. S4) downstream from the SCR may be involved in stabilizing the interaction between the SCR-proximal region and *Sox2* when the SCR is deleted. Cobound by CTCF, RAD21, and SMC1A (Fig. 1B), this site could act as a chromatin contact-anchoring region. Furthermore, given the functional redundancy between enhancer regions regulating *Sox2* transcription, it is plausible that CTCF-bound regions may also act redundantly to stabilize chromatin interactions. The dCTCF motif is oriented away from *Sox2* and not expected to participate in a direct interaction with the gene via stalled loop extrusion intermediates; however, other in silico data suggest that divergent CTCF pairing (such as that between SRR109 and dCTCF) cooperates to reinforce TAD and intra-TAD loops (Nanni et al. 2020). To evaluate this possibility, we created additional deletions at regions outside the SCR (Supplemental Fig. S6A). We extended the distal deletion to include the SCR and the downstream CTCF-bound region (ΔSCR-dCTCF/+), thus removing both CTCF-bound sites. However, these deletions demonstrated only a marginally increased loss of chromatin interaction (36%) compared with that observed with deletion of the SCR alone (28%), a variation in interaction frequencies that was not significantly different (*P* = 0.5) (Fig. 4B).

Upstream of the SCR, the locus contains two separate transcription factor-bound regions located 85 and 95 kb downstream from the *Sox2* promoter (SRR85 and SRR95) (Fig. 4A; Supplemental Fig. S4). These regions were previously shown to lack enhancer activity in a reporter assay (Zhou et al. 2014). Their deletion, either on their own (ΔSRR85–95/+) or in combination with the SCR (ΔSRR85–95+SCR-dCTCF/+), revealed that SRR85 and SRR95 also lack enhancer activity in their endogenous genomic context (Supplemental Fig. S6B). We tested whether these elements may play more of an architectural role in stabilizing a transcription factor-bound “hub” instead. Upon producing the compound deletion ΔSRR85–95+SCR-dCTCF/+, which removes all major CTCF and transcription factor-bound regions in and around the SCR, we surprisingly noted only a subtle further reduction (36%) in interaction frequency compared with deletion of the SCR alone (28%) (Fig. 4C). The extent of this reduction in chromatin–chromatin contacts appears identical to that caused by deletion of SCR to dCTCF alone, which would suggest that SRR85 and SRR95 play no additional role in chromatin architecture at this locus. However, we observed a reduction of interactions with regions just downstream from *Sox2* in ESCs lacking the SRR85 to SRR95 region (Fig. 4C, arrow).

When comparing all the locus deletions discussed thus far, we noted that the significant reduction in chromatin contacts at the *Sox2* locus was only achieved by deletion of the entire SCR; the combined deletion of SRR109 (and its resident CTCF site) and the two principal enhancer elements (ΔSRR107–111/+) (Fig. 3) caused only a minimal disruption in the interaction frequencies with the *Sox2* gene. We thus considered a possibility that the one remaining transcription factor-bound region in the SCR, SRR106, could safeguard the local chromatin architecture at the locus. To test this possibility, we generated an ESC clone combining deletions of all of the previously interrogated regions on the 129 allele but truncating the SCR to dCTCF deletion to leave SRR106 intact (ΔSRR85–95+107-dCTCF/+) (Fig. 4D). Distal interaction frequency with *Sox2* decreased by 43% in ΔSRR85–95+107-dCTCF/+ cells compared with wild-type cells (*P =* 0.01), yet this chromatin contact profile is quantitatively similar to and not different statistically from the reduction caused by deletion of the SCR (*P* = 0.3) (Fig. 4D). Thus, SRR106 does not appear to support the maintenance of local chromatin topology in the absence of other transcription factor-bound regions. Overall, these results reinforce the notion of a decoupling of *Sox2* transcriptional control and chromatin architecture within the locus. Whereas two single enhancer elements confer the vast majority of transcriptional control in pluripotent ESCs (at least under the conditions of the experiment), chromatin architecture supporting interactions between SCR-proximal regions and the *Sox2* gene is widely distributed over many contributing elements. Much of the chromatin contact profile (>50% of wild-type levels) was maintained in the absence of *Sox2* transcription and persisted even in cells with the most extreme deletions generated in this study (Supplemental Table S1).

### The downstream *Sox2* TAD border is insulated by the entire SCR in a CTCF-independent manner

**Figure 5.**
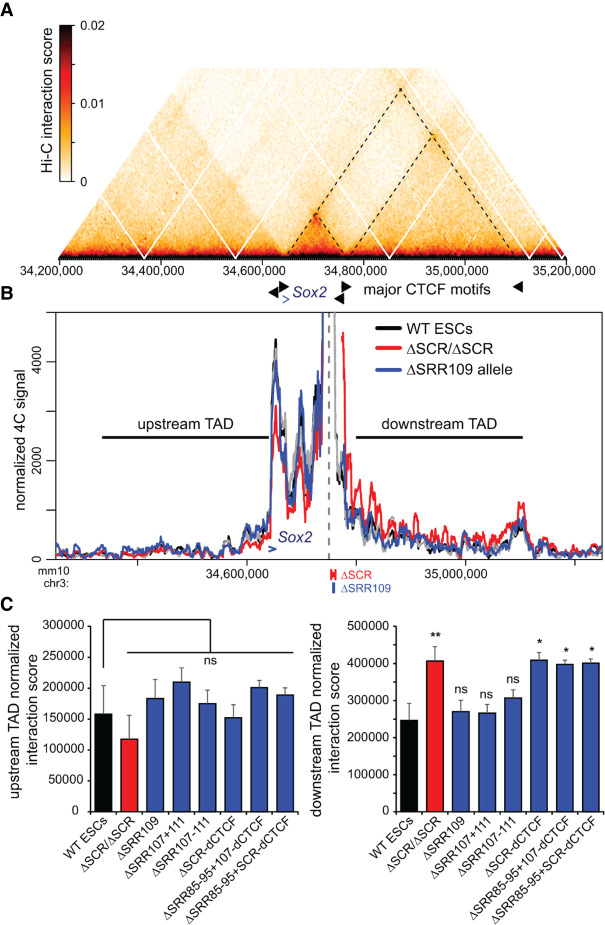
SCR deletion affects the *Sox2*–SCR TAD boundary and causes increased interaction with the downstream TAD. (*A*) Hi-C data from ESCs (acquired from Bonev et al. 2017) indicating the frequency of occurring interactions surrounding *Sox2*. The dashed lines correspond to the TAD boundaries at the *Sox2* promoter, the SCR, and the downstream TAD boundary. (*B*) 4C data are shown for wild-type cells (WT, black, *n* = 4), homozygous ΔSCR/ΔSCR cells (red, *n* = 4), and the ΔSRR109 allele in heterozygous ΔSRR109/+ cells (blue, *n* = 2). The dashed line indicates the location of the 4C bait region. The *Sox2* gene is indicated by a blue arrow. The horizontal lines indicate the 325-kb upstream and downstream TAD regions used to calculate the interaction score shown in *C*. For deletion alleles, the 4C signal has been omitted from the deleted region and flanking positions that are also affected by the deletion when computing running means. (*C*) Normalized interaction scores for the upstream (*left*) and downstream (*right*) TAD regions are shown for the indicated F1 ESC clones, revealing that only the full SCR deletion significantly increases interaction frequencies between the SCR proximal region and the downstream TAD. The blue bars mark the interactions observed for the deleted allele in the indicated heterozygous deletion-carrying clones. Significant differences from the interaction in WT cells are indicated. (*) *P* < 0.05, (**) *P* < 0.01, (ns) not significant. Data shown are an average of two to four biological replicates, with error bars representing SD.

Publicly available Hi-C data have shown that the *Sox2* gene and the SCR each reside near a TAD boundary, thereby restricting interactions with flanking chromatin outside the ESC-specific *Sox2* TAD (Fig. 5A; Supplemental Fig. S7A). When we evaluated interaction frequencies between the SCR-proximal bait region and genomic regions outside its resident TAD, we noted that removal of the SCR did not generate any specific ectopic interactions upstream of or downstream from the TAD borders, but did cause a general increase in 4C signal downstream from the SCR beyond the directly contiguous sequence (Fig. 1C, downstream from the peak marked by an asterisk). Extending the view of the 4C results to a larger window, it was clear by visual inspection that the SCR deletion caused a general increase in basal interaction frequency with the entire downstream chromatin up to the next TAD border, whereas interaction frequencies with the upstream TAD were unaffected (Fig. 5B). Although this type of boundary function is often associated with CTCF binding, we noted that removal of SRR109, which is the only region bound by CTCF within the SCR, does not disrupt the SCR boundary or cause any change in the contact profiles with either the upstream or downstream TADs. We next analyzed the interaction frequencies across the entire downstream TAD segment and the genomic region of the same size (325 kb) in the upstream TAD in ESCs harboring deletions of regions we had prioritized as candidate regulators of chromatin topology at the *Sox2* locus. We observed that only removal of the entire SCR—either alone or in combination with deletions of SRR85 and SRR95, and/or dCTCF—was associated with the observed “leakiness” of the downstream TAD border (Fig. 5C). No significant changes in interaction frequencies were observed with the upstream TAD, indicating that the effect is specific to the SCR TAD border and cannot be caused by normalization biases when intra-TAD interactions are reduced. This finding indicates that, similar to the *Sox2*–SCR interaction, the downstream TAD boundary is robustly maintained by multiple genomic elements acting in a partially redundant manner and is not conferred by the CTCF site alone.

Since the CTCF binding sites within the ESC *Sox2* TAD examined in this study appeared to be completely dispensable for local chromatin topology, we next asked whether the CTCF protein was required for *Sox2* expression and *Sox2*–SCR interaction. We reanalyzed Hi-C data from ESCs before and after acute CTCF depletion via an engineered auxin-inducible degron system, where large-scale disruption in TADs had previously been reported. Notably, RNA-seq showed only minor transcriptomic changes, including no effect on *Sox2* expression (Nora et al. 2017). In line with our own findings, CTCF ablation caused negligible (<1.04-fold) changes to the *Sox2*–SCR interaction frequency or overall *Sox2* TAD structure (Supplemental Fig. S7B), suggesting that local chromatin architecture is indeed CTCF-independent.

### *Sox2* transcription is maintained despite perturbation of chromatin contacts with the SCR

**Figure 6.**
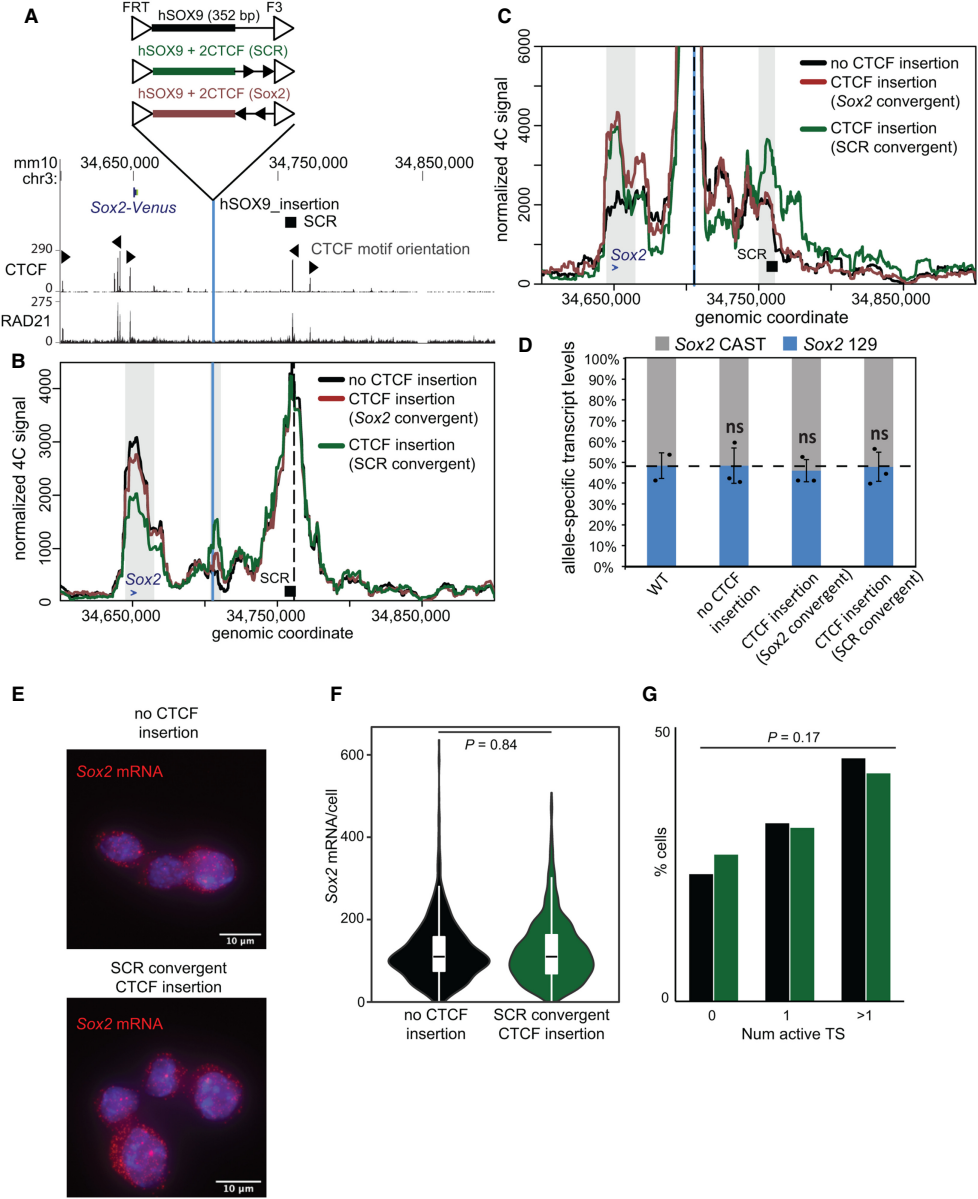
A CTCF motif insertion between the *Sox2* gene and the SCR disrupts the SCR–*Sox2* gene interaction, but not SCR-mediated enhancer activity. (*A*) The region surrounding the *Sox2* gene is displayed on the UCSC genome browser (mm10). The *Sox2* control region (SCR) is shown along with the orientation of the CTCF motifs within CTCF-bound regions. The vertical blue line represents the location into which the sequences shown *above* were integrated. CTCF and RAD21 ChIP-seq conducted in ESCs is shown *below*. In *B* and *C*, 4C data are shown for no CTCF integration (black), CTCF sites integrated in an orientation convergent with the SCR site (green), and CTCF sites integrated in an orientation convergent with the *Sox2* site (brown). The vertical blue line represents the location into which the sequences were integrated, and the dashed line marks the location of the 4C bait at the SCR (*B*) or the integration site (*C*). Gray boxes indicate the bait-interacting regions where significant differences were identified surrounding the *Sox2* gene (blue arrow), SCR (black box), or integration site (blue line). Compared with control cells lacking CTCF at the insertion site, a significant decrease in relative interaction frequency of the 4C bait region at the SCR with the *Sox2* gene was observed in the SCR-convergent CTCF insertion (*P* = 0.002), but not the *Sox2*-convergent CTCF insertion (*P* = 0.1). Compared with control cells, a significant increase in relative interaction frequency of the 4C bait region at the insertion with the *Sox2* gene site was observed in the SCR-convergent CTCF insertion (*P* = 0.02) and the *Sox2*-convergent CTCF insertion (*P* = 0.005). A significant increase in relative interaction frequency of the 4C bait region at the insertion with the SCR was observed in the SCR-convergent CTCF insertion (*P* = 0.02) but not the *Sox2*-convergent CTCF insertion (*P* = 0.6). (*D*) Allele-specific primers detect *Sox2 musculus* (129) or *castaneus* (CAST) mRNA by RT-qPCR from F1 ESC clones from the genotype indicated. Expression levels from either allele are shown relative to the total transcript levels. Error bars represent SD. *n* = 2–3. (ns) Not significant (*P* > 0.05). (*E*) Maximal projections of representative micrographs from smFISH experiments performed on ESCs with only the control sequence, or with the control sequence and CTCF sites convergent with the SCR, introduced at the insertion site. *Sox2* mRNA molecules are labeled in red, with DAPI staining in blue. Scale bar, 10 μm. (*F*) Violin plot comparing distributions of *Sox2* mRNA molecules per cell in ESCs with only the control sequence (black; *n* = 819 cells from two replicates), or the control sequence plus CTCF sites convergent with the SCR (green; *n* = 1289 cells from two replicates), at the insertion site. (*G*) Proportions of cells with zero, one, or more than one active transcription sites (TSs) for ESCs with only the control sequence (black), or the control sequence plus CTCF sites convergent with the SCR (green), at the insertion site.

The deletion experiments described thus far have allowed for the fine functional dissection of the SCR and SCR-proximal elements to evaluate the role these regions have in regulating *Sox2* expression and locus topology; however, these data did not indicate whether *Sox2* TAD architecture is in fact important for transcriptional control. All ESC lines harboring deletions that perturbed chromatin interactions had removed the SRR107 and SRR111 elements required for efficient *Sox2* expression. To assess the functional significance of the chromatin interactions at the *Sox2* locus, we chose to disrupt these endogenous *Sox2*–SCR interactions while keeping the SCR intact. We engineered an FRT/F3 cassette at a site located between the *Sox2* gene and the SCR in the 129 allele of F1 ESCs (Supplemental Figs. S8, S9; Supplemental Table S4). We were thus able to site-specifically insert putative insulator sequences of interest by recombinase-mediated cassette exchange and assess their effect on allele-specific chromatin topology and *Sox2* expression levels (Fig. 6A). This approach was previously applied to the *Sox2* locus; Huang et al. (2021) reported that efficient transcriptional insulation (i.e., reduction of *Sox2* expression levels by ∼30%–40%) required the insertion of tandem copies of CTCF motifs and flanking sequences (comprising ∼4-kb total inserted sequence) to alter endogenous *Sox2*–SCR interactions. In a parallel study to ours, Chakraborty et al. (2022) also demonstrated reduced *Sox2* expression in mouse blastocysts when tandem copies of CTCF motifs were homozygously inserted. However, these studies only assessed chromatin topology for insertions where significant transcriptional inhibition had been observed. Whether structural perturbations could occur in the absence of an effect on transcription had not been investigated.

We engineered a short human sequence insertion (352-bp DpnII–Csp6I fragment upstream of the *SOX9* gene), which could be used as a unique 4C bait. We introduced this insertion, with or without two copies of the core 19-bp CTCF motif within SRR109, into the landing site between *Sox2* and the SCR; the motifs were either both in convergent orientation with SRR109/SCR or with the CTCF site at the *Sox2* promoter (Fig. 6A). By ChIP-qPCR, we observed efficient recruitment of CTCF to the landing site only in the presence of the motifs (Supplemental Fig. S9E). With this setup, we were able to perform allele-specific 4C-seq using either the SCR (Fig. 6B) or the insertion site (Fig. 6C) as the 4C bait. Insertion of the human bait without accompanying CTCF sites had no effect on endogenous *Sox2*–SCR interaction frequencies. The insertion site did not interact with either the gene or the enhancer, although the observed abrupt loss of contact frequencies just distally of these elements was in line with their delimiting the *Sox2* TAD in ESCs (Fig. 5A). As may be expected from the loop extrusion model, inclusion of CTCF sites convergent with *Sox2* created an ectopic interaction between the insertion site and the gene (*P* = 0.005), but not with the SCR, from either the SCR or insertion site bait viewpoint (Fig. 6B,C; Supplemental Table S1). The endogenous *Sox2*–SCR interaction was unaffected (*P* = 0.1).

Inclusion of CTCF sites convergent with the SCR created an ectopic interaction between the insertion site and the SCR (*P* = 0.02), which was clearly detected from both SCR and insertion site bait perspectives (Fig. 6B,C). This profile was notably also accompanied by a 31% reduction in endogenous *Sox2*–SCR interaction frequencies, which is both significantly different from our control (*P* = 0.002) and quantitatively very similar to the interaction loss caused by deletion of the SCR. Surprisingly, the ectopic CTCF site also formed a strong and significant interaction with the *Sox2* promoter (*P* = 0.02)*,* which would not have been expected from a loop extrusion model due to the incompatible motif orientations at these regions. Contrary to other studies showing an effect on *Sox2* expression on insertion of larger CTCF-bound sequences (Huang et al. 2021; Chakraborty et al. 2022), we observed no allelic imbalance of *Sox2* expression in any of the tested insertion-carrying lines (Fig. 6D). Furthermore, single-molecule RNA FISH revealed no difference in overall *Sox2* mRNA levels or proportion of active transcription sites between cells lacking and cells carrying these CTCF site insertions, indicating similar bursting frequencies (Fig. 6E–G). Overall, these findings suggest that perturbation of chromatin architecture to the same extent as that caused by functionally significant enhancer deletions does not necessarily affect transcriptional output. Although stronger disruptions of chromatin–chromatin interactions have been shown to affect transcription (Huang et al. 2021), our data indicate that the loss of regulatory *cis* sequences at the SCR are directly responsible for *Sox2* transcriptional defects, and not the moderate loss of chromatin interactions that is also brought about by the deletion. Overall, this complementary approach reinforces the finding that the *Sox2*–SCR interaction appears very robust in ESCs, likely mediated by the contribution of many distributed elements, but that regulation of transcription and chromatin architecture can nonetheless be decoupled.

## Discussion

We have performed extensive allele-specific engineering at the mouse *Sox2* locus to functionally dissect the contributions of different regulatory elements to transcriptional regulation and modulation of chromatin architecture in ESCs. In so doing, we have uncovered a decoupling of the two processes. On the one hand, the vast majority of distal *Sox2* transcriptional regulation can be ascribed to a small number of transcription factor-bound regions, whereas maintenance of the 3D architecture of the locus is very robust and seemingly distributed over large regions within the TAD. Importantly, we were able to demonstrate that near-complete transcriptional shutdown by enhancer deletions had no effect on local chromatin topology, at least within the remit of what can be measured by population-averaged 3C-based approaches. The allele-specific application in our study was critical to dissecting the *cis* contributions of regulatory elements, since any small clonal variations were quantitatively controlled relative to the wild-type allele, and any potential confounding *trans* effects of loss of SOX2 protein were avoided (Huang et al. 2021; Vos et al. 2021).

Based on its relatively large size and clustering of binding sites for tissue-specific transcription factors, the SCR fits the canonical description of a “superenhancer” (Whyte et al. 2013). As such, the SCR is presumed to strongly activate target genes due to the combinatorial action of its composite transcription factor binding sites. However, the functional significance of “superenhancers” compared with nonclustered enhancers is markedly debated, with previous genetic dissections uncovering context-dependent transcriptional effects that may be functionally redundant or additive, but not synergistic (Hay et al. 2016; Moorthy et al. 2017; Saravanan et al. 2020; for discussion, see Blobel et al. 2021). Although the 7.3-kb-long SCR contains four regions, each bound by more than six different transcription factors, we found that only two of these regions (SRR107 and SRR111) account for the vast majority of *Sox2* transcription activation in *cis.* These two elements are each bound by eight or more transcription factors; however, their transcription-enhancing capacity can be greatly disrupted by removal of three motifs—an OCT4:SOX2 composite motif in SRR107 and two KLF4 motifs in SRR111—together totaling 37 nt. In contrast, chromatin architecture at this locus is not dependent on only a few nucleotides, as only deletions of the entire SCR or even larger regions displayed significant disruptions to the interaction frequencies with *Sox2* or the TAD boundary downstream from the SCR. The two enhancers within the SCR seem to be at least partially redundant, since disruption of both SRR107 and SRR111 is required for a strong transcriptional defect. None of the multiple other transcription factor-bound regions within or outside the SCR appear to offer any functional redundancy in ESCs from a transcriptional standpoint. Since the SCR adopts an inactive chromatin state and genome architecture in later developmental stages (Bonev et al. 2017; Ben Zouari et al. 2020), these regions are also not expected to act as enhancers in differentiated cells. However, since naïve state ESCs are an in vitro approximation of preimplantation epiblast cells (Evans and Kaufman 1981; Avilion et al. 2003), these elements may regulate *Sox2* expression in different in vivo pluripotent cell contexts. Alternatively, but not mutually exclusively, the “surplus” landing sites for transcription factors may indirectly facilitate the initiation of 3D folding of the locus during development to mediate transcriptional activation by the major regulatory elements.

A current question in the field is to what extent any interplay between CTCF-stalled cohesin-mediated loop extrusion and protein–protein interactions between transcription factors bound at distributed genomic sites affects chromosome folding, and whether this has any functional implications for enhancer activity (Dowen et al. 2014; Schuijers et al. 2018; Kubo et al. 2021). In this study, we assessed this question at the *Sox2* locus. The SCR contains one site within SRR109 that is strongly bound by CTCF and whose motif is oriented toward the CTCF sites just upstream of the *Sox2* promoter, rendering this site an ideal candidate mediator of promoter–enhancer interactions via stalled loop extrusion. However, in line with a previous study (de Wit et al. 2015), SRR109 is completely dispensable for *Sox2* transcription and SCR–*Sox2* interaction. Additionally, we show that removal of this single CTCF-bound region or acute depletion of CTCF protein is not sufficient to disrupt the TAD boundary downstream from the SCR, although deletion of the entire SCR does cause loss of topological insulation. Importantly, insertion of the SRR109 CTCF motif into other genomic locations is sufficient to induce ectopic chromatin loops; therefore, the absence of a corresponding phenotype from the SRR109 deletion is not due to a lack of the strength of the motif within this *cis* sequence. Instead, previous studies have shown a genomic context-dependent response of TAD borders to sequence deletions. Deletion of one or two CTCF sites at some loci, such as *Hoxa* and *Prdm14*, is sufficient for loss of TAD insulation and ectopic gene expression (Narendra et al. 2015; Vos et al. 2021), whereas other TAD borders, such as those at *Hoxd* and *Sox9/Kcnj2*, are highly resilient to large genomic deletions (Rodríguez-Carballo et al. 2017; Despang et al. 2019). At the *Sox2* locus, TAD border insulation is disrupted by the loss of the entire SCR, but not by deletion of its CTCF site, even when combined with the loss of both enhancers. We note that a parallel study prepared at the same time as our work also found the SCR CTCF site dispensable for both *Sox2* interaction and TAD border function in vivo (Chakraborty et al. 2022). Interestingly, this study additionally showed that the loss of the *Sox2* promoter-proximal CTCF sites had no effect on interaction frequencies with the SCR, but the TAD border upstream of the gene was perturbed. As well as further demonstrating the context-dependent nature of CTCF-mediated topological insulation, this apparent hierarchy of borders as “interaction attractors” could explain why an inserted intra-TAD CTCF site in either orientation ectopically interacted with the *Sox2* gene. The investigators for this study concluded that strong enhancer–promoter interactions could “bypass” topological insulation instructed by CTCF, but the CTCF-independent role of the SCR in TAD border maintenance was not assessed. We went further to show that the interaction and domain organization at *Sox2* are completely independent of CTCF and transcriptional activation.

With the negligible role of CTCF in orchestrating chromatin topology at the *Sox2* locus, other mechanisms could mediate enhancer–promoter interactions and/or TAD organization, such as cohesin recruitment at transcription factor-bound sites to initiate loop extrusion events (Liu et al. 2021; Vos et al. 2021), transcription factor and coactivator protein–protein interactions (Deng et al. 2012; Sabari et al. 2018), and ongoing transcription (Rowley et al. 2017; Hsieh et al. 2020). Strikingly, chromatin topology appears completely unaltered upon the compound deletion of SRR107 and SRR111, where transcription from the deleted allele is almost completely abolished. This result demonstrates a near-full uncoupling of transcriptional control and architecture maintenance by these two elements. Thus, ongoing transcription appears to have no role in *Sox2* chromatin topology, although a potential role for a low level of basal transcription cannot be ruled out. Instead, a role for transcription factor-bound sites themselves in mediating chromatin interactions is supported by a progressive, quantitative reduction of interaction as more transcription factor-bound regions are removed: no effect upon the deletion of SRR109 or SRR107 and SRR111; a weak, insignificant reduction upon removal of all sequences between and including SRR107 and SRR111 (including SRR109); a significant reduction upon loss of the entire SCR; and weak further losses of interaction frequencies when also removing transcription factor-bound regions at SRR85–SRR95 and/or an extension of the SCR deletion to a distal CTCF site. These data support the notion of a distribution of chromatin architecture maintenance over many genomic elements (presumably transcription factor-bound regions), within the *Sox2* TAD, each individually posing a weak effect but one that collectively builds up the domain. Since the cohesin complex subunit SMC1A is enriched at each of these contributing regions, the possibility is raised that cohesin is involved in *Sox2* locus topology maintenance. Alternatively, such a result is consistent with previous findings that distal regulatory elements can coassociate in nuclear “hubs” (Palstra et al. 2003; Allahyar et al. 2018; Oudelaar et al. 2018; Espinola et al. 2021), often involving clusters of specific transcription factors (Schoenfelder et al. 2010; Papantonis et al. 2012; Li et al. 2020a). Live-imaging experiments have identified such a cluster around the *Sox2* transcription site, comprising coassociated SOX2, BRD4, and RNA polymerase II (Li et al. 2020a). We thus propose that the ESC-specific *Sox2* TAD is established as a consequence of such clustering, delimited by the *Sox2* promoter and the SCR, which exhibit the greatest transcription factor site density. Other loci, such as *Pou5f1* (encoding OCT4), may be similarly coordinated (Li et al. 2020a), whereas imaging experiments support a predominantly CTCF-mediated mechanism for other tissue-specific chromosome domains, such as the mouse *α-globin* locus (Brown et al. 2018).

The requirement for spatial proximity of promoters and enhancers for transcriptional control has recently been questioned by imaging experiments showing gene transcription in the absence of enhancer coassociation (Alexander et al. 2019; Benabdallah et al. 2019). Whether the same observation applies to *Sox2* remains unclear. Consistent and large distances between *Sox2* and the SCR reported after imaging, when large operator sequences are inserted near the elements (Alexander et al. 2019), are not in agreement with frequent close proximities measured by DNA FISH in wild-type fixed cells (Huang et al. 2021). Our own and others’ studies identified an ESC-specific proximity between *Sox2* and the SCR as demonstrated in population-averaged 3C-based studies (Zhou et al. 2014; de Wit et al. 2015; Bonev et al. 2017). However, this is not sufficient proof for a causative link between chromatin contact and transcriptional regulation, not least because 3C-based studies cannot give insight into the potentially transient and dynamic nature of chromatin interactions, which may play an important functional role (Gabriele et al. 2022). Notably, none of our own or parallel attempts to disrupt SCR–*Sox2* interactions via ectopic CTCF-mediated loops (Fig. 6; Huang et al. 2021; Chakraborty et al. 2022) were able to completely abolish the endogenous promoter–enhancer interactions; therefore, none of these studies have been able to demonstrate definitively whether SCR-driven *Sox2* transcription can occur in ESCs with a complete absence of promoter–enhancer interactions. Conversely, perturbation of *Sox2* transcription required disruption of endogenous *Sox2*–SCR interactions at a higher level than that caused by deletion of the SCR. Taken together, the seemingly conflicting reports from 3C-based and imaging studies suggest at least some form of spatial proximity between promoter and enhancer at some point in the transcription cycle. Our single-molecule RNA FISH results do not provide evidence that moderately disrupted interactions result in a change in transcriptional bursting, suggesting that the residual interaction frequency is sufficient for full transcriptional firing. It has been proposed that hubs of transcription factors may generate unique nuclear microenvironments competent for transcription, perhaps within phase-separated condensates (Lim and Levine 2021). Such environments would facilitate interactions between genes and distal genomic elements; however, the increased local concentration of regulatory factors means that their juxtaposition would not be a strict prerequisite for transcriptional firing, but simply a coassociation within the same hub. Recent studies are beginning to dissect the relative requirements of phase separation and conventional protein–protein dimerization events in building these hubs (Chong et al. 2018; Li et al. 2020a; Wei et al. 2020).

Despite the attractiveness of the transcription factor hub model, it should be noted that even upon removal of six regions within the *Sox2* TAD, each bound by multiple transcription factors and accounting for at least 49 separate binding events, the domain structure appears robust. More than half of the detected interaction frequency between *Sox2* and the 4C bait adjacent to the SCR remains after these deletions, leaving open the possibility that additional mechanisms may contribute to local chromatin topology. We did note one additional region 15 kb downstream from the dCTCF site that is bound by OCT4, NANOG, and SOX2 in some but not all available ESC ChIP-seq data sets for these factors. This region could also be involved in supporting the additional interactions that persist after deletion of the other transcription factor-bound regions. Previous studies have shown that abolition of the *Sox2*–SCR TAD upon ESC differentiation to neuronal precursors (Bonev et al. 2017) was associated with a complete loss of promoter–enhancer interactions within only a few days of in vitro differentiation (Ben Zouari et al. 2020). These data suggest that any such mechanisms are still tissue-specific and not “hardwired” at this locus and are consistent with a role of the pluripotency-associated transcription factors in maintaining this topology. Specifically, the TAD border upstream of the *Sox2* promoter, with four promoter-associated CTCF sites, is maintained, but all topological insulation at the SCR is lost. Notably, the seemingly dispensable CTCF binding event at SRR109 is likewise lost (Bonev et al. 2017), but this is also accompanied by a down-regulation of the majority of pluripotency transcription factors that cluster at the SCR in ESCs (Dhaliwal et al. 2018). Combined with our own findings that local chromatin architecture is uncoupled from *Sox2* transcription and CTCF binding, the potential importance of spatial transcription factor clustering has not been fully established and warrants further investigation.

Overall, our results reveal that specific elements supplying transcriptional enhancer function contribute to, but are not required for, maintenance of chromatin structure. Instead, we identified a distributed role of multiple regulatory elements in organizing chromatin structure, in stark contrast to the small number of elements necessary for transcriptional regulation. The dispensability of CTCF motifs (and protein) in the *Sox2* locus for both chromatin interaction and boundary maintenance highlights the shortcomings of focusing on CTCF over other transcription factor binding events that can similarly contribute to promoter–enhancer interactions and TAD boundary maintenance.

## Materials and methods

### Cell culture

Mouse F1 ESCs (*M. musculus*^129^ × *M. castaneus*; female cells obtained from Barbara Panning) were cultured on 0.1% gelatin-coated plates in ES medium (DMEM containing 15% FBS, 0.1 mM MEM nonessential amino acids, 1 mM sodium pyruvate, 2 mM GlutaMAX, 0.1 mM 2-mercaptoethanol, 1000 U/mL LIF, 3 µM CHIR99021 [GSK3β inhibitor; Biovision], 1 µM PD0325901 [MEK inhibitor; Invitrogen]), which maintains ESCs in a pluripotent state in the absence of a feeder layer (Mlynarczyk-Evans et al. 2006; Ying et al. 2008).

### Cas9-mediated deletion

Cas9-mediated deletions were carried out as previously described (Zhou et al. 2014; Moorthy and Mitchell 2016). Cas9 targeting guide RNAs (gRNAs) were selected flanking the desired region identified for deletion (Supplemental Table S2). For select cases of allele-specific targeting, gRNA pairs were designed so that at least one gRNA overlapped a SNP to specifically target the *M. musculus*^129^ allele. On- and off-target specificities of the gRNAs were calculated as described in Doench et al. (2016) and Hsu et al. (2013), respectively, to choose optimal guides. Guide RNA plasmids were assembled with gRNA sequences using the protocol described by Mali et al. (2013). Briefly, two partially complementary 61-bp oligos were annealed and extended using Phusion polymerase (New England Biolabs). The resulting 100-bp fragment was assembled into an AflII-linearized gRNA empty vector (Addgene 41824) using the Infusion assembly protocol (TaKaRa Bio). The sequence of the resulting gRNA plasmid was confirmed by sequencing with either T7 or SP6 primers.

F1 ESCs were transfected with 5 µg of each of the 5′ gRNA(s), 3′ gRNA(s), and pCas9_GFP (Addgene 44719) (Ding et al. 2013) or pCas9_D10A_GFP (Addgene 44720) plasmids using the neon transfection system (Life Technologies). Forty-eight hours after transfection, GFP-positive cells were isolated on a BD FACSAria. Ten-thousand GFP-positive cells were seeded on 10-cm gelatinized culture plates and grown for 5–6 d until large individual colonies formed. Individual colonies were picked and propagated for genotyping and gene expression analysis as previously described (Zhou et al. 2014; Moorthy and Mitchell 2016). Genotyping of the deletions was performed by amplifying products internal to and surrounding the target deletion. All deleted clones identified from the initial screen were sequenced across the deletion; SNPs confirmed allele specificity of the deletion (Supplemental Table S3).

### Generation of insertion lines

A P2A-Venus reporter construct was inserted at *Sox2* on the *musculus* allele of F1 ESCs by homologous recombination after Cas9-mediated DNA break at the 3′ end of *Sox2*. A plasmid containing a P2A-Venus cassette and one containing Cas9-mCherry, a puromycin resistance gene, and three gRNA cassettes were assembled by the Institute of Genetics and Molecular and Cellular Biology (IGBMC) molecular biology platform (Supplemental Fig. S8A; vectors available on request). One gRNA targeted a Cas9-mediated DNA break at the 3′ end of *Sox2*, and the other two targeted breaks flanking the P2A-Venus cassette on the plasmid to generate 8-bp microhomology with the *Sox2* 3′ site (Supplemental Fig. S8A). Five micrograms of each plasmid was transfected into 1 million cells with Lipofectamine 2000, and Venus-positive cells were isolated by FACS after 5 d. Single clones were isolated, and incorporation of reporter into the *musculus* and/or *castaneus* allele was determined by allele-specific PCR. A *musculus*-incorporated heterozygous clone was further characterized by sequencing of PCR products. Expression of pluripotency markers, allelic *Sox2* expression, and 4C chromatin interaction profiles were unperturbed by incorporation of the Venus reporter (Supplemental Fig. S9).

This reporter line was then used for *musculus*-specific insertion of an FRT/F3 cassette into a site between *Sox2* and the SCR. One-kilobase homology arms were added to a plasmid containing a puromycin resistance–thymidine kinase positive selection marker within an FRT/F3 cassette (a kind gift from Marie-Christine Birling, Institut Clinique de la Souris) by restriction cloning (Supplemental Fig. S8B). Five micrograms of this plasmid was cotransfected with 5 µg of Cas9-mCherry/sgRNA plasmid (generated by the IGBMC molecular biology platform; vectors available on request) into 1 million cells with Lipofectamine 2000. Only the *musculus* allele had a functional PAM at the gRNA target site due to a SNP. Cherry-positive cells were isolated by FACS after 3 d, and after 1 d of recovery, clones were selected with 3 µg/mL puromycin for 1 d, and then 1 µg/mL puromycin until individual resistant colonies were formed. Clones were screened by PCR and confirmed by sequencing. We noted a slight reduction in *musculus*-specific *Sox2* transcription and SCR–*Sox2* interaction from this founder line, which was rescued on removal of the positive–negative selection marker (Supplemental Fig. S9).

The positive–negative selection marker was replaced with different inserts by FLP-mediated recombination. The donor vectors were constructed by restriction cloning of the initial FRT/F3 plasmid, annealed oligonucleotides (for CTCF motifs), and PCR products from human genomic DNA template (for SOX9 sequence). Five micrograms of donor vector was coelectroporated with 5 µg of FLP-expressing plasmid (a kind gift from Marie-Christine Birling, Institut Clinique de la Souris) with neon, and after 2 d of recovery, recombinant clones were selected with 6 µM ganciclovir. Clones were screened by PCR and confirmed by sequencing. Flow cytometry quantitation revealed a slight but equal reduction in Venus reporter expression in all clones compared with the founder line (Supplemental Fig. S9), even though allelic balance of *Sox2* expression was unaltered (Fig. 6D). ChIP-qPCR confirmed recruitment of CTCF to the insertion site only when cognate CTCF sites were present (Supplemental Fig. S9E).

### RNA isolation and gene expression analysis by RT-qPCR

Total RNA was purified from single wells of >85% confluent six-well plates using the RNeasy Plus mini kit (Qiagen), and an additional DNase I step was used to remove genomic DNA. RNA was reverse-transcribed with random primers using the high-capacity cDNA synthesis kit (Thermo Fisher Scientific). *Sox2* gene expression was detected by allele-specific primers that specifically amplified either the *musculus* or *castaneus* allele as described in Zhou et al. (2014) and Moorthy and Mitchell (2016). The standard curve method was used to calculate expression levels using F1 ESC genomic DNA to generate the standard curves. Levels of *Gapdh* or *Sdha* RNA were used to normalize expression values. Primer sequences are listed in Supplemental Table S5.

### Allele-specific 4C-seq

Cells were fixed with 2% paraformaldehyde in 10% FBS in PBS for 10 min at 23°C. The fixation was quenched with cold glycine at a final concentration of 125 mM, and then cells were washed with PBS and permeabilized for 1 h on ice with 10 mM Tris-HCl (pH 8), 100 mM NaCl, 0.1% NP-40, and protease inhibitors. Nuclei were resuspended in DpnII restriction buffer at 10 million nuclei/mL concentration (CutSMART buffer for *Sox2* promoter bait), and aliquots of 5 million nuclei were further permeabilized by treatment with 0.4% SDS for 1 h at 37°C and then incubated with 3.33% Triton X-100 for 1 h at 37°C. Nuclei were digested overnight with 1500 U of DpnII at 37°C (300 U of NlaIII for *Sox2* promoter bait) and then washed twice by centrifuging and resuspending in T4 DNA ligase buffer. In situ ligation was performed in 400 μL of T4 DNA ligase buffer with 20,000 U of T4 DNA ligase overnight at 23°C. DNA was purified by reverse cross-linking with an overnight incubation at 65°C with proteinase K, followed by RNase A digestion, phenol/chloroform extraction, and isopropanol precipitation. The DNA was digested with 5 U/μg Csp6I overnight at 37°C, and then repurified by phenol/chloroform extraction and isopropanol precipitation. The DNA was then circularized by ligation with 200 U/μg T4 DNA ligase under dilute conditions (3 ng/μL DNA) and purified by phenol/chloroform extraction and isopropanol precipitation. For allele-specific 4C from the SCR-proximal bait, samples of the DNA were digested with BveI or Alw26I, cutting specifically at the region between the DpnII site and the 4C reading primer annealing site on the 129 or *castaneus* allele, respectively. For 129-specific 4C from the SCR bait, the material was digested with AvaIII, cutting specifically at the region between the Csp6I site and the 4C nonreading primer annealing site on the *castaneus* allele. There were no SNPs allowing *castaneus*-specific 4C from this bait. No digestion was required for allele-specific 4C from the human insertion sequence, since this was only present on the 129 allele. 4C using the *Sox2* promoter as bait was not allele-specific, and no extra digestion step was incorporated. One-hundred-nanogram aliquots of treated DNA were then used as template for PCR with bait-specific primers containing Illumina adapter termini (primer sequences in Supplemental Table S6). PCR reactions were pooled, and primers were removed by washing with 1.8× AMPure XP beads and then quantified on a Bioanalyzer (Agilent) before sequencing with a HiSeq 4000 (Illumina).

### Western blotting

Protein was extracted from 5 million cells using RIPA buffer (50 mM HEPES-KOH, 500 mM LiCl, 1 mM EDTA, 1% NP-40, 0.7% Na-deoxycholate) containing Halt protease inhibitor complete EDTA-free (Thermo Fisher Scientific), supplemented with 1 mM PMSF and 2 mM Na_3_VO_4_. Total protein concentration was quantified using bicinchoninic acid (Thermo Fisher Scientific). Total protein samples were prepared in Laemmli buffer before being analyzed by SDS-PAGE (Bis-Tris; 4% stacking, 20% resolving). Blots were probed with primary antibodies for SOX2 (Cell Signaling Technology [CST] 23064) or UBF (sc-13125), followed by horseradish peroxidase-conjugated secondary antibodies. Blots were visualized with enhanced chemiluminescence.

### ChIP

Chromatin immunoprecipitation was performed as described previously (King and Klose 2017), with minor modifications. Forty million ESCs were cross-linked with 2 mM succinimidyl glutarate (Thermo Fisher Scientific) for 1 h at room temperature prior to cross-linking with 1% formaldehyde for 15 min with rotation. Reactions were quenched by adding glycine to a final concentration of 125 mM for 15 min with rotation. Cell pellets were washed twice with ice-cold PBS followed by resuspension in ice-cold lysis buffer 1 (50 mM HEPES-KOH, 140 mM NaCl, 1 mM EDTA, 10% glycerol, 0.5% NP-40, 0.25% Triton X-100) for 10 min at 4°C. Lysates were centrifuged at 2000*g* for 5 min at 4°C before resuspending in ice-cold lysis buffer 2 (10 mM Tris-HCl, 200 mM NaCl, 1 mM EDTA, 0.5 mM EGTA) for 10 min at 4°C. Nuclei were pelleted at 2000*g* for 5 min at 4°C before resuspending in ice-cold lysis buffer 3 (10 mM Tris-HCl, 100 mM NaCl, 1 mM EDTA, 0.5 mM EGTA, 0.1% Na-deoxycholate, 0.5% N-laurylsarcosine). Sonication was performed using a probe sonicator at 20 A (15 sec on/30 sec off) for 4.5 min at 4°C. After cell lysis and sonication, Triton X-100 was added to the sonicated lysate to precipitate any debris. Fifty microliters of cell lysate was obtained as whole-cell extract (WCE), and the remaining lysate was used for two immunoprecipitations. Chromatin samples were incubated overnight with 5 μg of relevant antibodies at 4°C with rotation. Antibodies used for ChIP experiments were anti-OCT4A (CST 5677), anti-SOX2 (CST 23064), anti-RNA polymerase II (Abcam ab5131), and anti-CTCF (Millipore 07-729). One-hundred microliters of protein A magnetic Dynabeads (Invitrogen) was added to the antibody-bound chromatin and incubated overnight at 4°C with rotation. Lysates were washed six times at room temperature with RIPA buffer followed by a TBS buffer wash. Samples were eluted with an SDS-based elution buffer (50 mM Tris-HCl, 10 mM EDTA, 1% SDS) for 30 min at 65°C before addition of 4 μL of 20 mg/mL proteinase K and overnight decross-linking at 65°C. ChIP DNA was purified using phenol/chloroform. Fold enrichment was calculated using the standard curve method with WCE used to generate the standard curve. For all ChIP experiments, except those pertaining to CTCF, allele-specific primers were used to specifically amplify either the *musculus* or *castaneus* allele as described (Zhou et al. 2014; Moorthy and Mitchell 2016). Primer sequences are listed in Supplemental Table S7.

### Single-molecule RNA fluorescent in situ hybridization (smFISH)

Three-hundred-thousand cells were seeded onto poly-L-lysine-coated coverslips in culture medium and incubated for 2–3 h at 37°C and 5% CO_2_ to attach to the coverslips. Cells were washed three times with warm Hank's buffered salt solution (Gibco), fixed with 4% paraformaldehyde for 10 min, and washed twice for 10 min in PBS. The cells were permeabilized with 70% ethanol overnight at 4°C. Coverslips were hybridized overnight at 37°C with hybridization buffer containing 10% dextran sulfate, 10% formamide, 2× SSC, and 5 pmol of fluorescent probes targeting *Sox2* and labeled with Quasar670 dye (Biosearch Technologies). Coverslips were washed three times for 30 min with 10% formamide and 2× SSC at 37°C, once with 2× SSC, and once for 5 min with PBS at room temperature. Coverslips were mounted on microscope slides using ProLong Gold mounting medium containing DAPI (Thermo Fisher). Coverslips were dried for at least 24 h at room temperature in the dark before imaging. Imaging was performed on an inverted microscope (Zeiss AxioObserver), a plan-apochromat 40× 1.4 NA oil DIC UV objective, a 1.60× optovar, and an sCMOS camera (Hamamatsu Orca Flash 4v3). For Quasar670, a 660-nm longpass dichroic (Chroma T660lpxrxt), 697/60-nm emission filter (Chroma ET697/60 m), and 640/30-nm LED excitation at full power (Spectra X, Lumencor) were used. For DAPI, a 425-nm longpass dichroic (Chroma T425lpxr), a 460/50-nm emission filter (Chroma ET460/50 m), and LED excitation at 395/25 nm at 25% power (Spectra X, Lumencor) were used. For each sample and each channel, we used the Micro-Manager software to acquire at several fields of view, each consisting of a 45-*z*-stack (Δ*z* 0.3 μm) at 25-msec exposure for DAPI and 750-msec exposure for Quasar670.

### 4C-seq analysis

Sequencing read fastq files were demultiplexed with sabre (https://github.com/najoshi/sabre) and aligned to the mm10 genome with Bowtie (Langmead et al. 2009), and intrachromosomal reads were assigned to DpnII or NlaIII fragments by utility tools that come with the 4See package (Ben Zouari et al. 2020). 4See was also used to visualize the 4C profiles. Interactions were called for each replicate with peakC (Geeven et al. 2018) with window size set to 21 fragments, and were then filtered to only include the regions called as interacting across all wild-type replicates. We note that the “minimal” region spanning *Sox2* was called as interacting with the proximal SCR bait for all cell lines (and virtually all replicates) tested in this study (Supplemental Table S1). For statistical comparison of specific interactions, the 4C read counts within 1 Mb of the bait for all replicates and conditions (from the same bait) were quantile-normalized using the limma package (Ritchie et al. 2015). The means of summed normalized 4C scores over tested interacting regions were taken as “interaction scores” and were compared across conditions by two-tailed *t*-tests. For the SCR–*Sox2* and insertion site–*Sox2* interactions, we used the minimal region spanning *Sox2* for all wild-type replicates with the near-SCR bait (mm10; chromosome 3: 34,644,922–34,664,967). For the insertion site–SCR interaction, we used the minimal region spanning the SCR called as interacting in all replicates of the (CTCF insertion [SCR-convergent]) line (mm10; chromosome 3: 34,749,652–34,760,919). For quantifying inter-TAD interactions from the near-SCR bait, we used the 325-kb region (mm10; chromosome 3: 34,800,000–35,105,000) that contains nearly the entire downstream TAD. Since 4C-seq signal at the 5′ end of the downstream TAD is artificially inflated in various deletion lines because the genomic separation has been shortened by the deletion, the selected region started at a conservatively chosen place 3′ to 4C-seq local minima (i.e., when the contact decay with genomic separation had equilibrated for all the cell lines). As a control for the upstream TAD, the same size region was used (mm10; chromosome 3: 34,315,000–34,640,000).

### Hi-C reanalysis

Published Hi-C data (Bonev et al. 2017; Nora et al. 2017) were downloaded from GEO (GSM2533818–2533821 for ESC DpnII, GSM2533822–2533825 for NPC DpnII, GSM2644945–2644946 for ESC control HindIII, and GSM2644949–2644950 for ESC CTCF degron HindIII) and reanalyzed using the FAN-C toolbox (Kruse et al. 2020), entailing read-mapping, filtering out technical artifacts, mapping to restriction fragment space, binning, matrix normalization, ratio-based comparison, and visualization.

### smFISH analysis

A custom Python script was used to detect, localize, and classify the spots (https://github.com/Lenstralab/smFISH). Cells and nuclei were segmented using Otsu thresholding and watershedding. Spots were localized by fitting a 3D Gaussian mask after local background subtraction (Coulon et al. 2014) and counted per cell. Cells in which no spots were detected were excluded from further analysis, since a visual inspection indicated that these cells were not properly segmented. The numbers of RNAs at any cluster were determined by normalizing to the median fluorescent intensity of the cytoplasmic RNAs detected in all cells. Nuclear foci containing ≥2.5 RNAs were classified as active transcription sites (TSs), and the numbers of cells containing zero, one, or more active TSs were counted (a small number of cells appeared to have more than two active TSs due to imperfect focus calling). The two technical replicates were found to be extremely similar and were pooled for statistical analysis. Distributions of mRNA counts per cell were compared by Wilcoxon rank sum test, and the proportions of cells with no, monoallelic, or biallelic transcriptional firing were compared by χ^2^ test.

### ChIP-seq visualization

ESC ChIP-seq data sets and associated peak files were obtained from the CODEX database (Sánchez-Castillo et al. 2015).

### Data availability

All 4C-seq data from this study have been deposited on GEO with the accession GSE195906.

## Supplementary Material

Supplemental Material
