# Supplementary material for: Transcriptional regulation and chromatin architecture maintenance are decoupled functions at the Sox2 locus
Source: Genes Dev. 2022 Jun 1;36(11-12):699–717. doi: 10.1101/gad.349489.122 (PMC9296009; doi:10.1101/gad.349489.122)
Supplement: Supplemental Material [file supp_36_11-12_699__DC1.html]

Transcriptional regulation and chromatin architecture maintenance are decoupled functions at the Sox2 locus — Transcriptional regulation and chromatin architecture maintenance are decoupled functions at the Sox2 locus — Supplemental Material 

# Transcriptional regulation and chromatin architecture maintenance are decoupled functions at the *Sox2* locus

## Supplemental Material

- Supplemental\_FigS1.pdf
- Supplemental\_FigS2.pdf
- Supplemental\_FigS3.pdf
- Supplemental\_FigS4.pdf
- Supplemental\_FigS5.pdf
- Supplemental\_FigS6.pdf
- Supplemental\_FigS7.pdf
- Supplemental\_FigS8.pdf
- Supplemental\_FigS9.pdf
- Supplemental\_TableS1.xlsx
- SupplementalTables.docx
