# Supplementary material for: Transcriptional regulation and chromatin architecture maintenance are decoupled functions at the Sox2 locus
Source: Genes Dev. 2022 Jun 1;36(11-12):699–717. doi: 10.1101/gad.349489.122 (PMC9296009; doi:10.1101/gad.349489.122)
Supplement: Supplemental Material [file supp_gad.349489.122_Supplemental_FigS1.pdf]

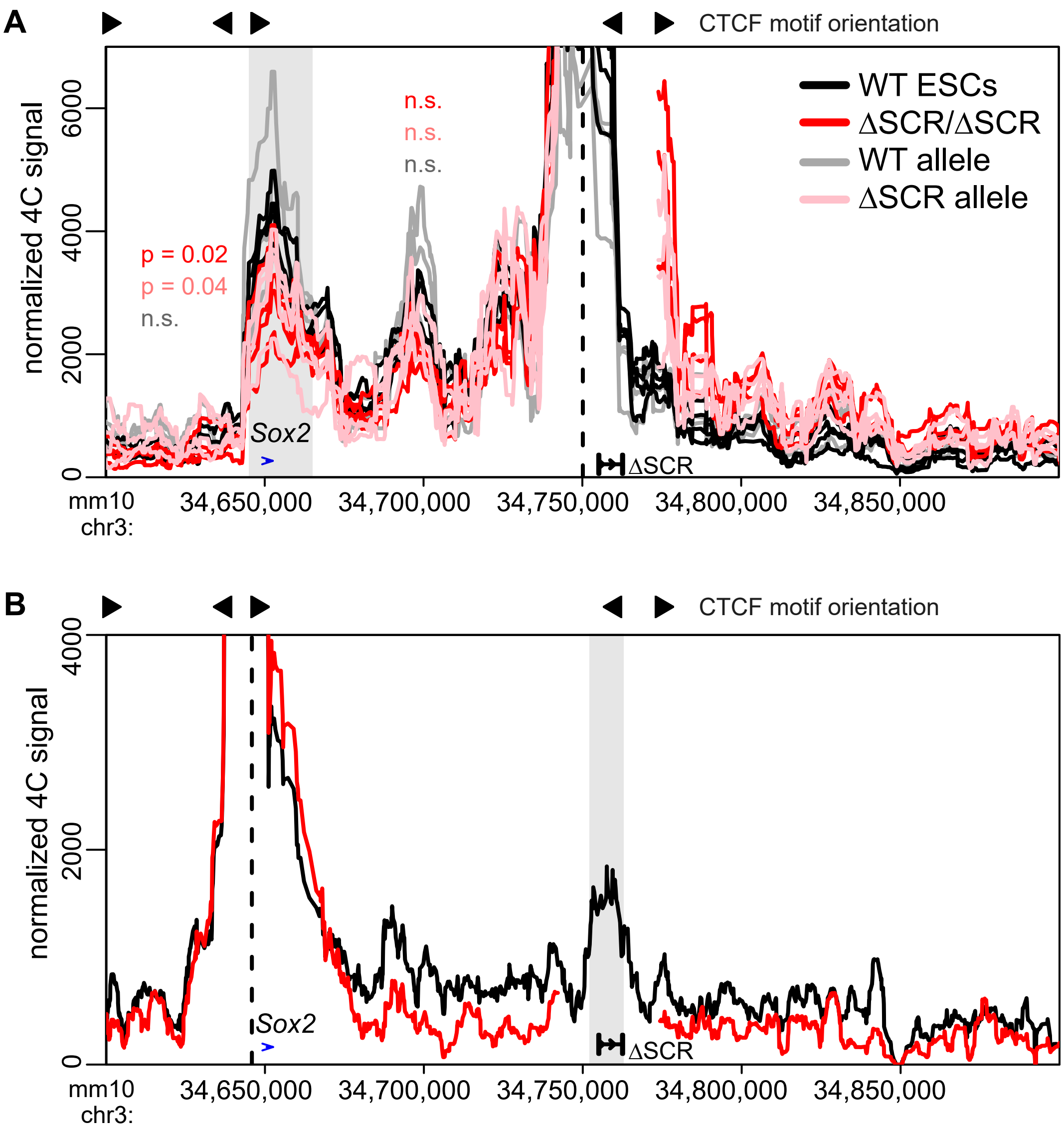

Figure S1. Validation of *Sox2*-SCR interaction. A) 4C data (running mean) are shown for wild-type (black, n=4), homozygous ΔSCR/ΔSCR cells (red, n=4) and heterozygous ΔSCR cells. Data from the heterozygous cells are displayed separately for the WT (grey, n=3) and ΔSCR alleles (pink, n=4). The data are the same as for **Fig 1C**, but replicates are plotted individually instead of being averaged for one profile. The dashed line indicates the location of the 4C bait region. The grey box indicates the bait-interacting region surrounding the *Sox2* gene. The interaction with the gene is the only called interaction that shows statistically significant differences between wild-type and ΔSCR (p-values for comparison with homozygous WT are indicated, colour-coded according to genotype; n.s. indicates $p > 0.05$). Apparent differences in interaction strength with an intervening region between *Sox2* and the SCR are not reproducible across replicates (denoted on plot). For deletion alleles, the 4C signal has been omitted from the deleted region and flanking positions that are also affected by the deletion when computing running means. B) 4C data (running mean) are shown for wild-type (black, n=2) and homozygous ΔSCR/ΔSCR cells (red, n=1) with the bait proximal to the *Sox2* promoter, confirming the *Sox2*-SCR interaction in wild-type ESCs. The dashed line indicates the location of the 4C bait region. The grey box indicates the called bait-interacting region surrounding the SCR. For the deletion line, 4C signal has been omitted from the deleted region and flanking positions that are also affected by the deletion when computing running means. Positions of the *Sox2* gene, deleted region and CTCF-bound sites, along with the orientation of the CTCF motifs, are shown in both plots.
