# Supplementary material for: Transcriptional regulation and chromatin architecture maintenance are decoupled functions at the Sox2 locus
Source: Genes Dev. 2022 Jun 1;36(11-12):699–717. doi: 10.1101/gad.349489.122 (PMC9296009; doi:10.1101/gad.349489.122)
Supplement: Supplemental Material [file supp_gad.349489.122_Supplemental_FigS2.pdf]

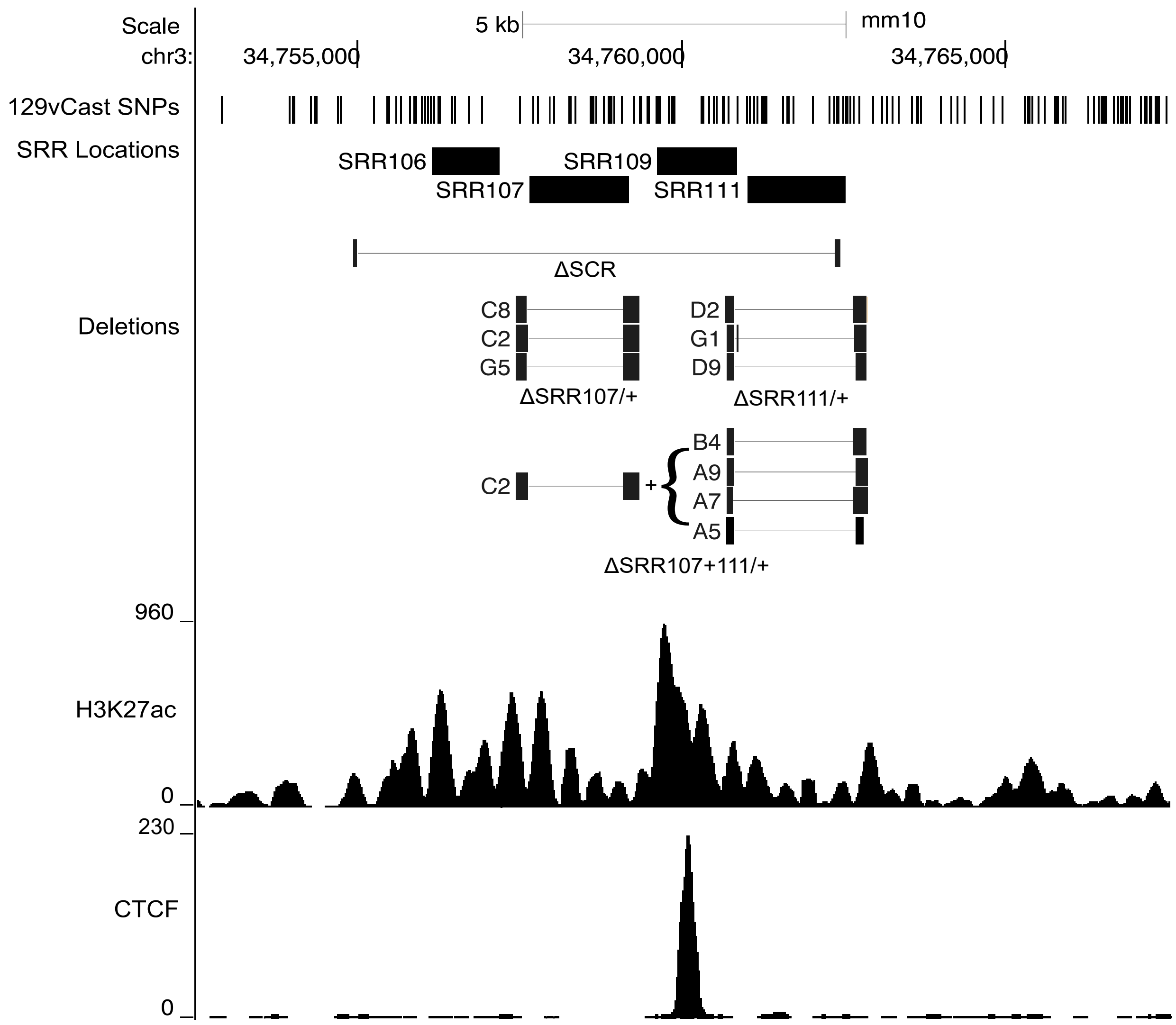

Figure S2. Details on the generation of sub-SCR deleted cell lines targeting highly transcription factor-bound regions. Schematic representation of SCR region along with deletion boundaries displayed on the University of California Santa Cruz (UCSC) Genome Browser (mm10). Schematics explained from top to bottom: Genome coordinates, positions of Mus castaneus SNPs, positions of the SCR and SRR sub regions and the sequenced clones harboring the specified deletion, ChIP-seq of H3K27ac and CTCF in wild-type mouse ESCs. For compound deletions ΔSRR107+111/+ clones were created in the background of ΔSRR107/+ C2. SCR deletion is shown as a comparison to the listed clones.
