# Supplementary material for: Transcriptional regulation and chromatin architecture maintenance are decoupled functions at the Sox2 locus
Source: Genes Dev. 2022 Jun 1;36(11-12):699–717. doi: 10.1101/gad.349489.122 (PMC9296009; doi:10.1101/gad.349489.122)
Supplement: Supplemental Material [file supp_gad.349489.122_Supplemental_FigS3.pdf]

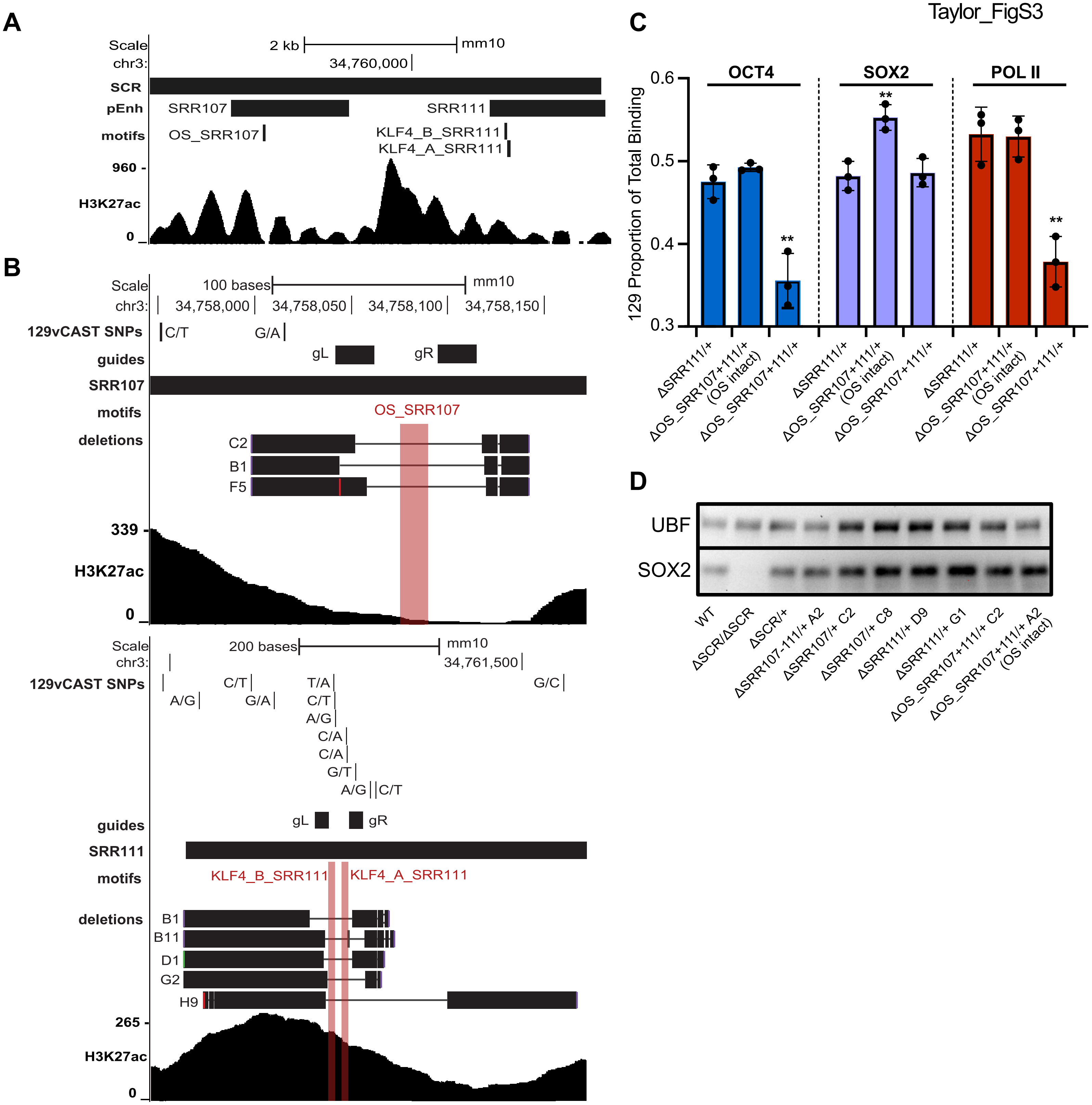

Figure S3. Details on the generation of sub-SCR deletions targeting specific transcription factor motifs. A) Schematic representation of high-scoring predictive transcription factor motifs located in SRR107 or SRR111 of the SCR. SRR107 contains a high-scoring OCT4:SOX2 motif (OS_SRR107), while SRR111 contains two high-scoring KLF4 motifs (KLF4_A_SRR111 and KLF4_B_SRR111, respectively) displayed on the UCSC Genome Browser (mm10). B) Resulting 129-specific deletions of targeted motif deletions are shown. Schematics explained from top to bottom: Genome coordinates, positions of Mus castaneus SNPs, positions of the gRNAs, SRR sub region, the sequenced clones harboring the specified deletion, ChIP-seq of H3K27ac in wild-type mouse ESCs. The positions of the targeted transcription factor motifs are shown in red. C) Chromatin immunoprecipitation followed by qPCR (ChIP-qPCR) was performed to detect binding of OCT4, SOX2, or RNA Polymerase II nearby the high-scoring OCT4:SOX2 motif located in SRR107. Relative binding was quantified and shown using allele-specific primers to detect the proportion of protein binding near the 129-specific deletion relative to total binding across both alleles. Error bars represent SD, n = 3. Significant differences from the parent clone (SRR111/+) values are indicated: (*) P < 0.05, (**) P < 0.01, (***) P < 0.001, (****) P < 0.0001, ns = not significant. D) Western blot on multiple SCR sub deleted clones reveals no loss of SOX2 protein expression among heterozygous SCR and SCR sub deletions. UBF is the loading control.
