# Supplementary material for: Transcriptional regulation and chromatin architecture maintenance are decoupled functions at the Sox2 locus
Source: Genes Dev. 2022 Jun 1;36(11-12):699–717. doi: 10.1101/gad.349489.122 (PMC9296009; doi:10.1101/gad.349489.122)
Supplement: Supplemental Material [file supp_gad.349489.122_Supplemental_FigS4.pdf]

Taylor_FigS4

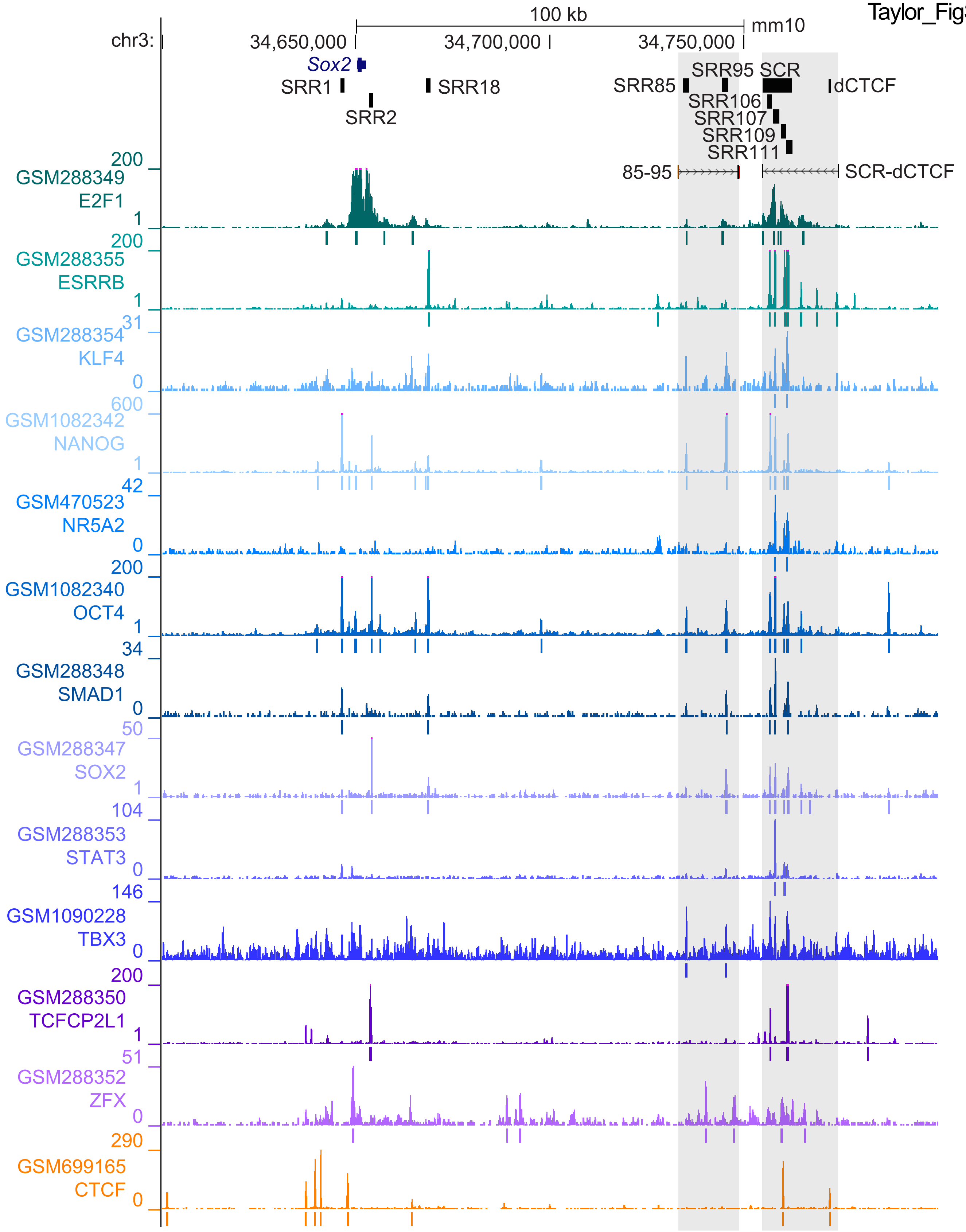

Figure S4. Expanded view of transcription factor binding across the *Sox2* locus. The region surrounding the *Sox2* gene is displayed on the UCSC Genome Browser (mm10). SRRs correspond to transcription factor-bound regions derived from ESC ChIP-seq datasets compiled in the CODEX database. Called ChIP-seq peaks are located and color coded beneath their requisite tracks. The region corresponding to the deletions of SRR85 to SRR95 and of SCR to the distal CTCF-bound region (ΔSRR85-95+ SCR-dCTCF/+) is highlighted in grey overlapping the sequenced deletion boundaries.
