# Supplementary material for: Transcriptional regulation and chromatin architecture maintenance are decoupled functions at the Sox2 locus
Source: Genes Dev. 2022 Jun 1;36(11-12):699–717. doi: 10.1101/gad.349489.122 (PMC9296009; doi:10.1101/gad.349489.122)
Supplement: Supplemental Material [file supp_gad.349489.122_Supplemental_FigS5.pdf]

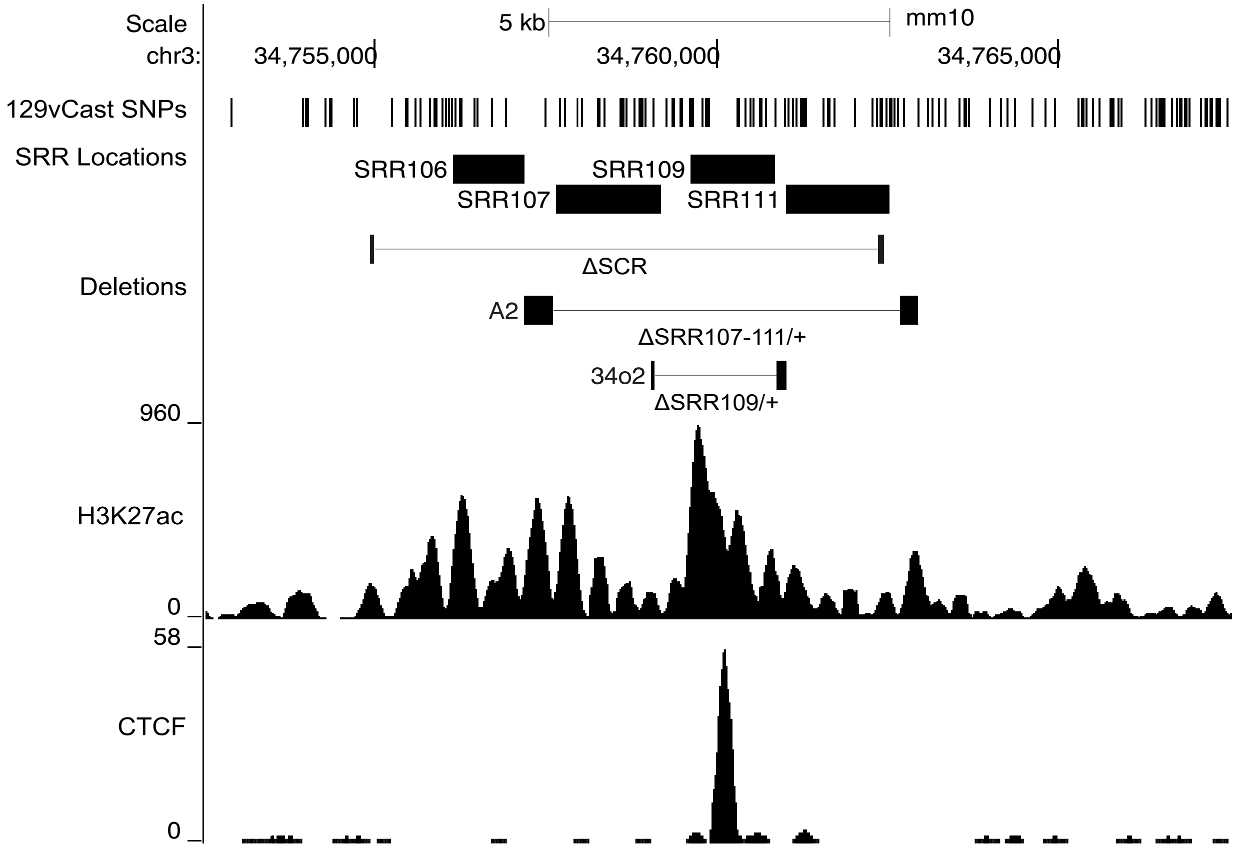

Figure S5. Details on the generation of sub-SCR deleted cell lines targeting the central CTCF-bound region and surrounding transcription factor-bound sites. Schematic representation of the deletion encompassing both enhancer regions (SRR107 and SRR111) and the CTCF-bound peak at SRR109, as well as the SRR109 region alone within the SCR, displayed on the UCSC Genome Browser (mm10). Schematics explained from top to bottom: Genome coordinates, positions of Mus castaneus SNPs, positions of the SCR and SRR sub-regions, the sequenced clones harboring the specified deletion, ChIP-seq of H3K27ac and CTCF in wild-type mouse ESCs. SCR deletion is shown as a comparison to the listed clones.
