# Supplementary material for: Transcriptional regulation and chromatin architecture maintenance are decoupled functions at the Sox2 locus
Source: Genes Dev. 2022 Jun 1;36(11-12):699–717. doi: 10.1101/gad.349489.122 (PMC9296009; doi:10.1101/gad.349489.122)
Supplement: Supplemental Material [file supp_gad.349489.122_Supplemental_FigS6.pdf]

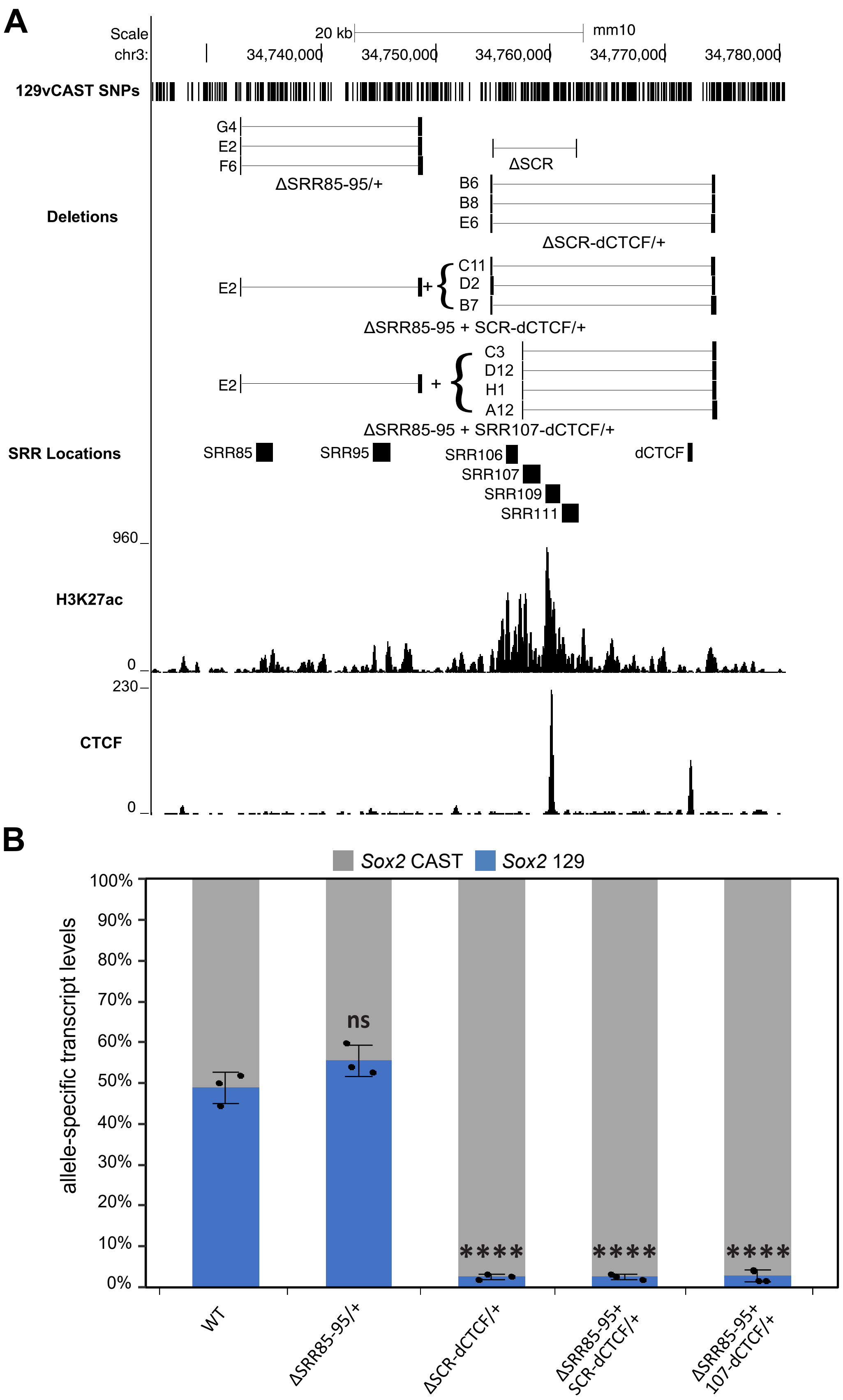

Figure S6. Details on the generation of deletion lines including targets surrounding the SCR. A) Schematic representation of the regions targeted for larger 129-specific deletions removing the SCR alongside a downstream CTCF-bound region displayed on the UCSC Genome Browser (mm10). Schematics explained from top to bottom: Genome coordinates, positions of Mus castaneus SNPs, positions of the SCR and SRR sub regions, the sequenced clones harboring the specified deletion, ChIP-seq of H3K27ac and CTCF in wild-type mouse ESCs. For compound deletions, clone ΔSRR85-95/+ E2 was used to create ΔSRR85-95+SCR-dCTCF/+ and Δ SRR85-95+107-dCTCF/+. SCR deletion is shown as a comparison to the listed clones. B) *Sox2* expression in wild-type F1 cells (WT) compared to clones carrying the indicated deletion on the 129 allele. Allele-specific primers detect musculus (129) or castaneus (CAST) RNA in RT-qPCR. Expression levels are normalized to transcript levels from GAPDH. Error bars represent the SD. Significant differences between wild-type cells and clones denoted by (*), with adjusted p-value <0.0001 (****), <0.001 (***), <0.01 (**), <0.05 (*) or non-significant (n.s.).
