# Supplementary material for: Transcriptional regulation and chromatin architecture maintenance are decoupled functions at the Sox2 locus
Source: Genes Dev. 2022 Jun 1;36(11-12):699–717. doi: 10.1101/gad.349489.122 (PMC9296009; doi:10.1101/gad.349489.122)
Supplement: Supplemental Material [file supp_gad.349489.122_Supplemental_FigS7.pdf]

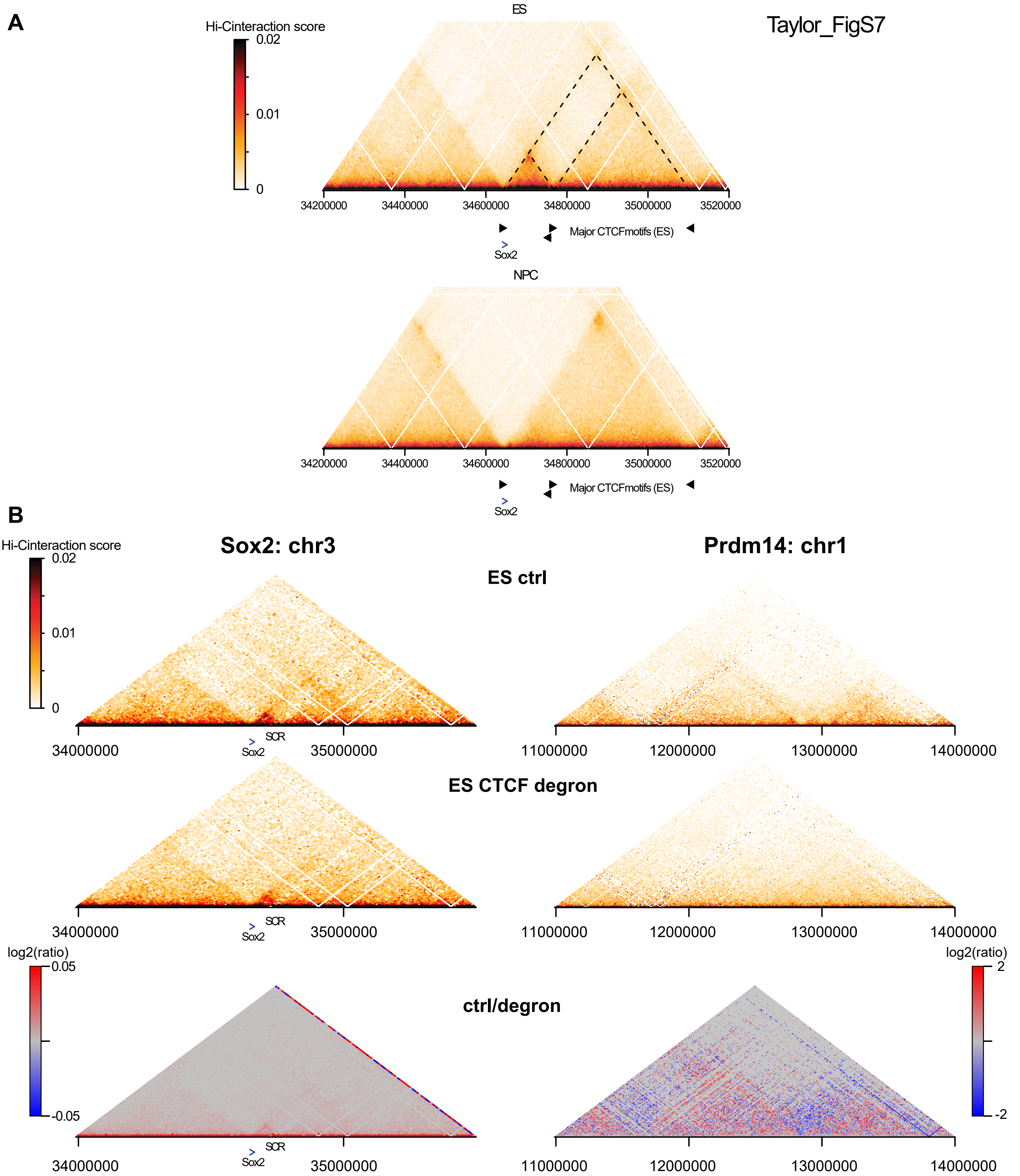

Figure S7. Chromatin architecture around the *Sox2* locus is ESC-specific and CTCF-independent. A) Hi-C maps around the *Sox2* locus for ESCs (top) and neuronal precursor cells (NPC, bottom), generated from data from Bonev et al. (2017). Positions and orientations of the *Sox2* gene and major CTCF-bound motifs in ESCs are denoted under the maps. Dotted lines indicate ESC TADs. These two TADs fuse into one larger TAD in NPCs. B) Hi-C maps around the Sox2 locus (left) and a control region around the *Prdm14* locus (right) in control ESCs (top) and ESCs after acute depletion of CTCF using an engineered auxin-inducible degron (middle); generated from data from Nora et al. (2017). Whereas clear loss of TADs on CTCF depletion is observed in the control region, *Sox2* architecture appears largely unchanged. Bottom: Quantitative comparison of the two experimental conditions at these regions is shown as a heatmap of log2(ctrl/degron) interaction scores. In the control region, CTCF depletion causes a ~4-fold relative increase in inter-TAD contacts and a similar decrease in intra-TAD contacts. At *Sox2*, contact changes are negligible (<1.04-fold).
