# Supplementary material for: Transcriptional regulation and chromatin architecture maintenance are decoupled functions at the Sox2 locus
Source: Genes Dev. 2022 Jun 1;36(11-12):699–717. doi: 10.1101/gad.349489.122 (PMC9296009; doi:10.1101/gad.349489.122)
Supplement: Supplemental Material [file supp_gad.349489.122_Supplemental_FigS8.pdf]

Taylor_FigS8

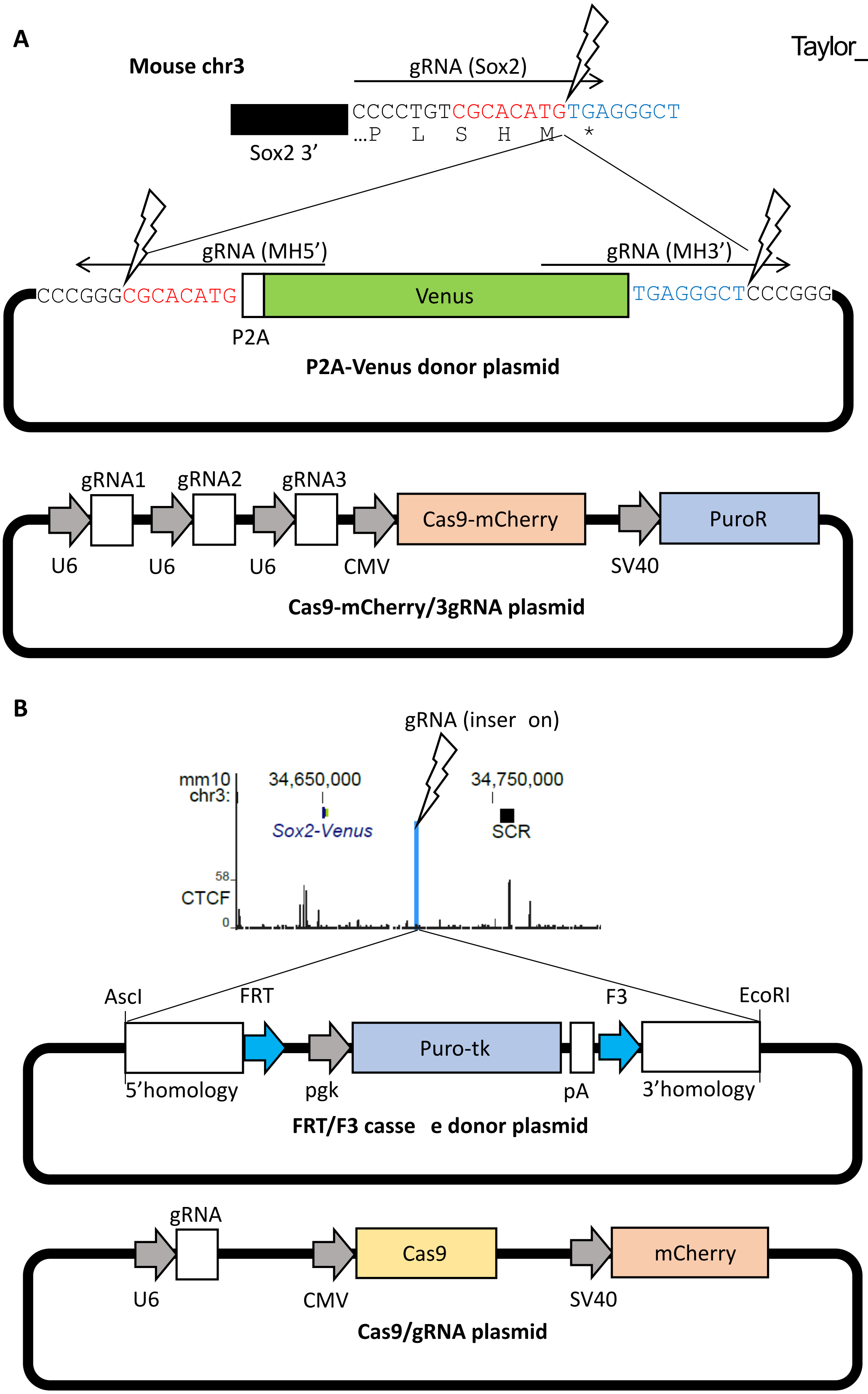

Figure S8. Construction of *Sox2* insertion lines. A) Plasmids constructed to generate the Venus tag at the 3' end of *Sox2*. F1 ESCs are transfected with a plasmid containing the P2A-Venus cassette (middle) and a plasmid (bottom) with Cas9-mCherry, a puromycin resistance marker and expression constructs for three gRNAs: *Sox2*, which targets CRISPR/Cas9 to the 3' end of the *Sox2* coding sequence (top), and MH5' and MH3', which target CRISPR/Cas9 to the termini of the P2A-Venus cassette to generate 8 bp microhomology arms. B) Insertion of the recombinase-mediated cassette exchange construct. The Sox2-Venus+/- line is transfected with two plasmids: one (middle) containing an FRT-puro-tk-F3 cassette for positive-negative selection, flanked by homology arms for the *Sox2* intervening sequence, and one (bottom) containing Cas9, mCherry and a construct for expression of one gRNA, which targets CRISPR/Cas9 to a musculus site located between *Sox2* and the SCR (top).
