# Supplementary material for: Transcriptional regulation and chromatin architecture maintenance are decoupled functions at the Sox2 locus
Source: Genes Dev. 2022 Jun 1;36(11-12):699–717. doi: 10.1101/gad.349489.122 (PMC9296009; doi:10.1101/gad.349489.122)
Supplement: Supplemental Material [file supp_gad.349489.122_Supplemental_FigS9.pdf]

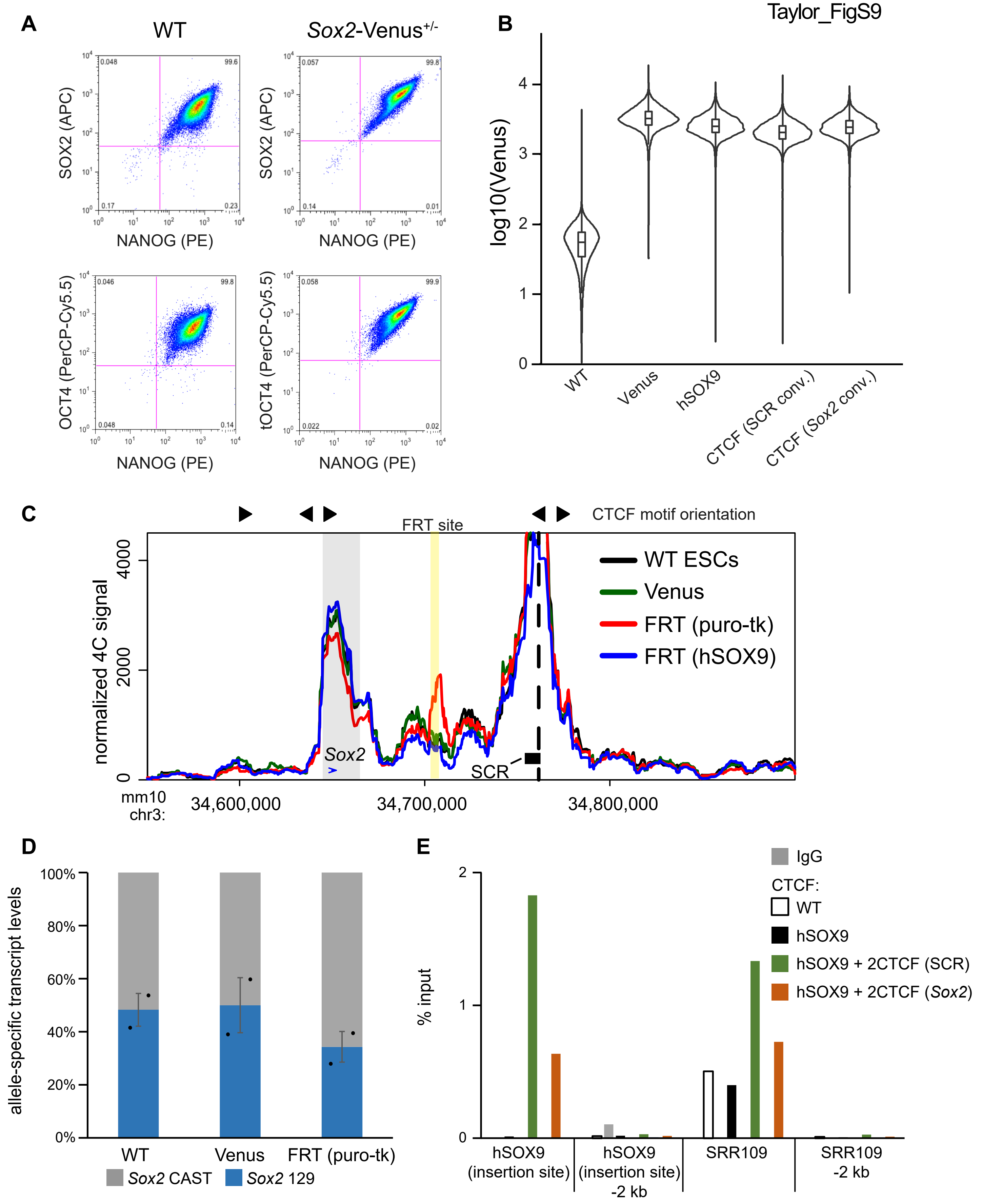

Figure S9. Characterization of the *Sox2* insertion lines. A) Flow cytometry profiles of F1 (left) and Sox2-Venus+/- (right) ESCs after staining with labeled antibodies to SOX2, NANOG and OCT4, showing that stemness is unaffected on insertion of the Venus reporter tag. B) Flow cytometry quantification of Venus fluorescence in the ESC lines with different insertions. Venus reporter is highly and equivalently expressed from lines where the hSOX9 tag, with or without CTCF sites, is inserted between *Sox2* and the SCR, which is slightly lower than the founder Sox2-Venus+/- line. C) Musculus-specific 4C profiles using the SCR as bait (dashed line) for F1 (black), Sox2-Venus+/- (green), FRT/F3/positive-negative selection marker (red), and FRT/F3/hSOX9 (blue) lines. The *Sox2*-SCR interaction is largely maintained, with a slight decrease in the presence of the positive-negative selection marker. D) Allele-specific qPCR quantification of *Sox2* expression, relative to SHDA, for the musculus (129; blue) and castaneus (CAST; grey) alleles in F1, Sox2-Venus+/-, and FRT/F3/positive-negative selection marker lines. Musculus expression is weakly reduced in the presence of the selection marker. Error bars show SD (n=2) (n.s. = non-significant). E) ChIP-qPCR on the insertion lines shows that CTCF is specifically recruited to the landing site when cognate motifs are present; CTCF is always detected at the endogenous SRR109 region.
