# Supplementary material for: Transcriptional regulation and chromatin architecture maintenance are decoupled functions at the Sox2 locus
Source: Genes Dev. 2022 Jun 1;36(11-12):699–717. doi: 10.1101/gad.349489.122 (PMC9296009; doi:10.1101/gad.349489.122)
Supplement: Supplemental Material [file supp_gad.349489.122_SupplementalTables.docx]

**SUPPLEMENTARY TABLES**

**Supplementary Table S1:** **Called interactions for each 4C-seq replicate experiment performed in this study.** Provided as a separate Excel file.

**Supplementary Table S2: Guide RNA sequences for CRISPR/Cas9 mediated deletions.**

∆SCR guide RNAs are shown for clarity but were originally designed in Zhou *et al.,* 2014

| **Target Region** | **Left Guide Sequence** | **Right Guide Sequence** | **Use with Cas9 or Cas9-D10A (nickase)** |
| --- | --- | --- | --- |
| ∆SCR | TAGCATACGTCACGCCGGAA | ACTGTTCTCGAACACTCTGT | Cas9 |
| ∆SRR107-111 | GACAAAAACATGTACGTTGGG | GGCCAAGGTTGAGCTCTAGT | Cas9 |
| ∆SRR107 | GACAAAAACATGTACGTTGGG | CATTCCTTGGCCAGATGCTA | Cas9 |
| ∆SRR111 | CTTAAATTTTATTTTGTGCT | CTTGCTGAAGAGAACTAACC | Cas9-D10A |
|  | TGGTCCCAGCATGTGCATA | GGCCAAGGTTGAGCTCTAGT |  |
| ∆SRR109 | CATTCCTTGGCCAGATGCTA | GAGTTGAAAAGATGGCTCAG | Cas9-D10A |
|  | ACATTGAACTAAGATCATTT | CTTAAATTTTATTTTGTGCT |  |
| ∆Oct:Sox_107 | TAGTCCCAGGACTCTGCTAA | GGTGGGTAGTTAGCATAATG | Cas9-D10A |
| ∆Klf4(x2)_111 | AGAAGATGAGATGAAAGGCA | TTGAAGGCAGCCTTCCGGTA | Cas9-D10A |
| ∆SRR85-95 | AACTTAGTGGACCATACCCA | CAGTATGACACGCAGTGGCG | Cas9 |
| ∆SCR-dCTCF | TAGCATACGTCACGCCGGAA | GCTGCAAAGGCTCCCGTTCG | Cas9 |
| ∆SRR107-dCTCF | GAAGACAAAAACATGTACGT | GCTGCAAAGGCTCCCGTTCG | Cas9 |

**Supplementary Table S3: Sequences across CRISPR/Cas9 mediated deletions.**

Removed region is denoted with a vertical line where CRISPR/Cas9 double-strand break or nick sites have been joined together. ∆SCR clones were created in Zhou *et al.,* 2014. All sequences are from the 129 allele except for specified ∆SCR clones. Targeted regions are also listed by their coordinates from the UCSC genome browser build mm10 based on gRNA locations.

| **Deletion Target (mm10)** | **Clone** | **Included in 4C Analysis (Y/N)** | **Deletion Sequence** |
| --- | --- | --- | --- |
| ∆SCR/∆SCR(Cast)  chr3: 34754958- 34762355 | 1 | Y | AACTATAATTTCCTGTACAGTCTTTCTTTAGACAGGGCTTCTGCTGCCTCTGAGTGGAAGATTGCTGGGATCATATGCCATCATATACATACCTGCACATATATGTGGGCACTTGCTGTAGGCAGAGGCCAGAAGAGGACAACACATCCCCTGGCTAGAGTTACAGTCAGCTCTGGAAAAAGCAGTAAGTGCTCTAACCACTGAGCTACCGTTCCG**\|**TAGTCAGGGATGCACAGAGAAACCGTCTCAAAAGACAAATACAGTAACCAAGACCAAAACCAACAACCAAACCAAACCAAACACAAACCCCAAAACAAAAGGCTAAGCTATTCC |
| ∆SCR/∆SCR (129)  chr3: 34754958- 34762355 | 1 | Y | CCCCTGGAAAAGCAGTAAGTGCTCTAACCACTGAGCTACCGTTC**\|**GAGTGTTCGAGAACAGTCAGGGATGCACAGAGAAACCGTCTCAAAAGACAAATACAATAACCAAGACCAAA |
| ∆SCR/+ (129)  chr3: 34754958- 34762355 | 15 | Y | TCCCCTGGAAAAGCAGTAAGTGCTCTAACCACTGAGCTACCGTTC**\|**GAGTGTTCGAGAACAGTCAGGGATGCACAGAGAAACCGTCTCAAAAGACAAATACAATAACCAAGACCACCAAC |
| +/∆SCR (Cast)  chr3: 34754958- 34762355 | 11 | Y | ATTTAGAATTTTTAAAGAATTTTTATTTTATTTTTAATTTATTTTTTACACTTCATATTCCACTCCCTAACCCCCCATCATTTAGAATTTTAATGGATTTCCTTCTATATATTTTTATTAATTTTTTCTGTTTTAATTTTTTCTTTTTAAATTTTTCTTGTTTAATCTTTCTTAAATTCAATTTAAAAATTTTTAACATAAATAACTAATTTGTGTTAGTTATATGTAAACAGCGGCTTTCCCTGTGTCATTTTCAGGCGTTTGTGTAGAGCATT**\|**CAAAAGAGGGGGGGAAATGTAGATAATAATAAGAATAACTAATTCCTCCTACGTTCACCCTCTAAAGGTAACAGTTATCTTTGTGGTTCTGAGCCTCCGGAGACTTGGGACGAACTTCACAGCCCTGGGTGTGTGGGCTCTATGCTTTCTCCTGAAATGTGGTTCATGGTTAGCTTGGGTTTGGGTTGCTACTTTGAAGAAATAAAGCAGGCTGGGTATGGTAGCACAGGCCTTTAAACCCAACATTCCAAAGGCTGAGGCAGACAGATGCATCTCTGGGAGGAGGAGGCCAGCCTGGTAACACAGTTAAATAAATCCTGGCGCACACACACAGAACTATTTACTACATTGCGGAGAGAGTAGAGGCTACGGAAGACCTGAAGTACCACCTGCCCTTGTTTTGGGACAAGACGTCACACTGTATCCCAGGATGGCCTGGAATTCACTATGTAAACTAGGTTGACTTTGAATTTGCAATGATCTTCCTTCCTCTACCTCCTGCCAAGATTATTAGGCATGGCCCACCACACCTGGCTCACTCATTTTAGTATCCTGGAGTATTGTACCGCATACAAACCCTAATATATATATAATATTTGGTTGCAGGAGGTGGGTTGTGGGCCCATGTGTGAGAAGACAACTTGTCGGGGCTAGTTCTCTCCCTCAGCCATGTGGATTCTGGGGCCTGAACCCAGGTCTGCAAGCAAGTGGCCAGCATCTTTCCTTGCAGACTCTGCCGCACACTGATTTTTTTGAGGGGGGTGGGGTGGGGTGAGACGGGGGGGAAGGACCTTGATATATAGTTCTCTACTGGCTTAGAACACCCTTGTGTCAGGAATAGTGACACTCACGCCTTTAAACCTAGCACTCGGGAGGCAGAGGCAGGCAGATTTCTGAGTTTGAGGCCAGCCTGGTCCATAGAGTGAGTTCCAGGACAGCCAGGGCTATACAGAGAAACCCTGTCTCAAAAAAAAAAAAAAAAAAAAAAACTCCTTCTATACATCAGGCTGGCTCCAACTTGCAGAGATCTGCTTGCCTCTGCCCTCTGCGCGAGTGCTGGAACTACAGGCATGCACAACACGCCCAGCCTCAGACTCAGCCTCTTAATCAAATGCACAGAAATAAGTTGGATGCTTCTCTTGAGATGTTGCCTAAGTATATATGGATATACTTACTCATTTATTGCTATTGGGAAAGACTCTGCTCTGGTACAGTTACAGTGACTCACAATTATAATTCCAGTACTGAGGAGTATGAGGGAAAGGAATTGCTTCAGGTAAGAAGTTACCCTGGGCTATACAACAAGATTCCATCTTGAACCTCCCTCTTTTACACTGAAAGGAAGTCCGGGGTGATTGCTAGCAGGAAACTTTTCTGATTGAAAGACAGCCCAAAGCTAGTAAGTCACACACTTCAGTCACGGGTGCAAAGTCCCCCAGGTAACTGACACTGGTCACTTCTTAGGAACCATTGAGTCAGGGAAAGAATCAATCTGAGTGTATACATATAGCATACGTCA\|TACGTGGTTAGAGCACTTACTGCTTTTTCCAGAGCTGACTGTAACTCTAGCCAGGGGATGTGTTGTCCTCTTCTGGCCTCTGCCTACAGCAAGTGCCCACATATATGTGCAGGTATGTATATGATGGCATATGATCCCAGCAATCTTCCACTCAGAGGCAGCAGAAGCCCTGTCTAAAGAAAGACTGGTACAGGAAAATGCAGAGTTAATGTAATGGGGTGGAGGAACAAGAGTCGGGGGCGGGGGGGGGGGGGGGGGTATCTCAGGCCTGAAGTCCCAAGAATCAAGAAGCTGAGGCAGGAGAATAGTCTCCAGCTTGAGGTCAGCCCAGGTTAAAAATGACAGCTTGTAGAGAATGGGAGAAGAAGAGAAATTAATGGTTGGTTAATATGATCAC\|GTTCGAGAACAGTCAGGGATGCACAGAGAAACCGTCTCAAAAGACAAATACAGTAACCAAGACCAAAACCAACAACCAAACCAAACCAAACACAAACCCCAAAACAAAAGGCTAAGCTATTCCTCCTAACTCTTAGCATTGCTCAAAGTGTGCCTGCTGTCCTAGGGGCAGGCCCTGGGCCAGATTCACCCCGGTTAAGCAGAATATATGTCAGGTCTCCTGTGGGCCAGAGATTTTGCAGATGAGGTGTTTTTGACTCTAACAGTATAGTACCAGACTGTCATCTCAGCACTTAGGAGGCTGAGGCAGGGGGATTGCAATGAGTTCCAGGTTAGTTCTCTTCAGTGAGACCCTGATTCAAAACCAAAACAAGGCCAAGGTTGAGCTCTAGTTGGCAGAATGCTCATCAGGTAGTTCTTGGAGGCTTTGAATCTGGAATCCCCTAGCCTCAGCCTTCCAGGAGCTAGATTACAAGCAAGTGCTGTTTCTATTAAATACCATGTTTTGGGTTTGGGGAGATGGGCCAGTGGTCAAGAGTGCTTGCTTCTCCTTCAGTGGACCAGACAGACTTCAGTTCCCAGCATCCAGGTTGGGTGGTCCACAGCCCCCTAACATTCCAGCTTCAGGGGAATCTGATACCCTCT |
| ∆SRR107-111/+  chr3:34757618-34762637 | A2 | Y | CCCTGAGCTGGATGTTAGGGGGCTGTGGACCACCCAACCTGGATGCTGGGAACTGAAGTCTGTCTGGTCCACTGAAGGAGAAGCAAGCACTCTTGACCACTGGCCCATCTCCCCAAACCCAAAACATGGTATTTAATAGAAACAGCACTTGCTTGTAATCTAGCTCCTAGAAGGCTGGGGCTAGGGGATTCCAGATTCAAAGCCTCCAAGAACTACCTGATGAGCATTCT**\|**ATGGGGGACTTTTGGTATAGCATTGGAAATGTAAATGAGCTAAATACCTAATAAAAAATGAAAAAAAAATGTACGTTTAAACTCAAAATCATGATGTCATGATGATGAAGTGCTGGGGGAGCAAGACAGGGCCATTGCCAGGGTAGCCTGTGCTACAGAATAAGACCCGGTCTCAAAAGAGAGCGCCGAGGGTGGGGGAACTTCCCTACGCCATCATCCCCCCCACACCCCTCCCCCAAAGAGGGGGAAAAGGCAAAAAAGCAGTAAATAAGAGATAGTCTAGTGGTACCGCCTGTCATCCCAGCTACTCAGGAAGCTAAGGCAGTTACCTGATGAGTGTAAGGCCTGCCTGGGCTACATGGGTTCAAAGCTAACCCTGGGCAACTTACTGAAACTCTTTCAAAAGATAAAAAAGAGAACTGG |
| ∆SRR107/+  chr3:34757618-34759104 | C2 | N | AATAATTGAGGCCATGCTAGTCTACAGATTGAGTTCCAGGACAGGCCGGGATGCACAGAGAAACCCTGTCTTGAAAACACCCCCAAAAAATCAATCCAGTAGATGGAATAAAGTATTTTGTGAATGATCTCAAACTCCTGATTGTCAAGGTTCCCCTGGCATGAATGGTCTTTATTGTTAATAATAGTTCTTCTCGTGATCAAATATATACCTAAATGATCTTAGTTCAATGTATCATTCCTTGGCCAGATG**\|**GGGTGGGTATGGGGGACTTTTGGTATAGCATTGGAAATGTAAATGAGCTAAATACCTAATAAAAAATGAAAAAAAAATGTACGTTTAAACTCAAAATCATGATGTCATGATGATGAAGTGCTGGGGGAGCAGAGACAGGGCCATTGCCAGGGTAGCCTGTGCTACAGAATAAGACCCGG |
|  | C8 | N | ACTGGATATTAAATAATTGAGGCCATGCTAGTCTACAGATTGAGTTCCAGGACAGGCCGGGATGCACAGAGAAACCCTGTCTTGAAAACACCCCCAAAAAATCAATCCAGTAGATGGAATAAAGTATTTTGTGAATGATCTCAAACTCCTGATTGTCAAGGTTCCCCTGGCATGAATGGTCTTTATTGTTAATAATAGTTCTTCTCGTGATCAAATATATACCTAAATGATCTTAGTTCAATGTATCATTCCTTGGCCAGATG**\|**GTATAGCATTGGAAATGTAAATGAGCTAAATACCTAATAAAAAATGAAAAAAAAATGTACGTTTAAACTCAAAATCATGATGTCATGATGATGAAGTGCTGGGGGAGCAGAGACAGGGCCATTGCCAGGGTAGCCTGTGCTACAGAATAAGACCCGG |
|  | G5 | N | GCACTGGATATTAAATAATTGAGGCCATGCTAGTCTACAGATTGAGTTCCAGGACAGGCCGGGATGCACAGAGAAACCCTGTCTTGAAAACACCCCCAAAAAATCAATCCAGTAGATGGAATAAAGTATTTTGTGAATGATCTCAAACTCCTGATTGTCAAGGTTCCCCTGGCATGAATGGTCTTTATTGTTAATAATAGTTCTTCTCGTGATCAAATATATACCTAAATGATCTTAGTTCAATGTATCATTCCTTGGCCAGATG**\|**TGGGTATGGGGGACTTTTGGTATAGCATTGGAAATGTAAATGAGCTAAATACCTAATAAAAAATGAAAAAAAAATGTACGTTTAAACTCAAAATCATGATGTCATGATGATGAAGTGCTGGGGGAGCAGAGACAGGGCCATTGCCAGGGTAGCCTGTGCTACAGAATAAGA |
| ∆SRR109/+  chr:34759104-34760865 | 34o2 | Y | AAATAAAAGATTAAGCAGTGTGATATACAAGCCTCTAAGACACACATAAAATGAACCTTATTGCTTATTGAAACTGGGTCTTATTATACAGCCAAGGCTAGCCCAGCACAAAATAAAATTTAAGATGGAAATT**\|**GGCTCAAACCTGTAATCCCGACTCTGGAAGCTGATTGTTT |
| ∆SRR111/+  chr:34760865-34762618 | D2 | N | ATATGTCTTATTATTTATTTTATGAGTATGAATATTTTGCCTGTTTGTATGTCTGTGCACCATGTGCATGCCTGGTGCTAATGGAGGCCAGAGGAGGGCATCAGGCCCTCTGGAGCTAGAGTTACAGATGGTTGTGAGCCTCT**\|**AAAACCAAAACAAGGCCAAGGTTGAGCTCTAGTTGGCAGAATGCTCATCAGGTAGTTCTTGGAGGCTTTGAATCTGGAATCCCCTAGCCCCAGCCTTCTAGGAGCTAGATTACAAGCAAGTGCTGTTTCTATTAAATACCATGTTTTGGGTTTGGGGAGATGGGCCAGTGGTCAAGAGTGCTTGCTTCTCCTTCTAGTGGACCAGACAGACT |
|  | D9 | N | ATGAATATTTTGCCTGTTTGTATGTCTGTGCACCATGTGCATGCCTGGTGCTAATGGAGGCCAGAGGAGGGCATCAGGCCCTCTGGAGCTAGAGTTACAGATGGTTGTGAGCCTCTATGCACATGCTGG**\|**GCAGAATGCTCATCAGGTAGTTCTTGGAGGCTTTGAATCTGGAATCCCCTAGCCCCAGCCTTCTAGGAGCTAGATTACAAGCAAGTGCTGTTTCTATTAAATACCATGTTTTGGGTTTGGGGAGATGGGCCAGTGGTCAAGAGTGCTTGCTTCTCCTTCAGTGGACCA |
|  | G1 | N | AATATTTTGCCTGTTTGTATGTCTGTGCACCATGTGCATGCCTGGTGCTAATGGAGGCCAGAGGAGGGCATCAGGCCCTCTGGAGCTAGAGTTACAGATGGTTGTGAGCCTCTA**\|**AACCAAGTTGGCAG**\|**AATGCTCATCAGGTAGTTCTTGGAGGCTTTGAATCTGGAATCCCCTAGCCCCAGCCTTCTAGGAGCTAGATTACAAGCAAGTGCTGTTTCTATTAAATACCATGTTTTGGGTTTGGGGAGATGGGCCAGTGGTCAAGAGTGCTTGCTTCTCCTTCAGTGGACCAGACA |
| ∆SRR107+111/+  chr3:34757618-34759104  +  chr:34760865-34762618 | A5 | Y | GAGTATGATATTTTGCCTGTTTGTATGTCTGTGCACCATGTGCATGCCTGGTGCTAATGGAGGCCAGAGGAGGGCATCAGGCCCTCTGGAGCTAGAGTTACAATGGTTGTGAGCCTCTA**\|**TTGGCAGAATGCTCATCAGGTATTCTTGGAGGCTTTGAATCTGGAATCCCCTACCCCACCTTCTAGGACTAATTACAACAATGCTGTTTCTATTAAATACCATGTTTTGG |
|  | A7 | N | ATTTTGCCTGTTTGTATGTCTGTGCACCATGTGCATGCCTGGTGCTAATGGAGGCCAGAGGAGGGCATCAGGCCCTCTGGAGCTAGAGTTACAGATG**\|**AAAACCAAAACAAGGCCAAGGTTGAGCTCTAGTTGGCAGAATGCTCATCAGGTAGTTCTTGGAGGCTTTGAATCTGGAATCCCCTAGCCCCAGCCTTCTAGGAGCTAGATTACAAGCAAGTGCTGTTTCTATTAAATACCATGTTTTGGGTTTGGGGAGATGGGCCAGTGGTCAAGAGTGCTTGCTTCTCCTTCAGTGGACCAGACAGACTTCAGTTC |
|  | A9 | N | ATTTTGCCTGTTTGTATGTCTGTGCACCATGTGCATGCCTGGTGCTAATGGAGGCCAGAGGAGGGCATCAGGCCCTCTGGAGCTAGAGTTACAGATGGTTGTGAGCCTCT**\|**GTTGGCAGAATGCTCATCAGGTAGTTCTTGGAGGCTTTGAATCTGGAATCCCCTAGCCCCAGCCTTCTAGGAGCTAGATTACAAGCAAGTGCTGTTTCTATTAAATACCATGTTTTGGGTTTGGGGAGATGGGCCAGTGGTCAAGAGTGCTTGCTTCTCCTTCAGTGGACCAGACAGACTTCAGTTC |
|  | B4 | N | TGAATATTTTGCCTGTTTGTATGTCTGTGCACCATGTGCATGCCTGGTGCTAATGGAGGCCAGAGGAGGGCATCAGGCCCTCTGGAGCTAGAGTTACAGATGGTTGTGAGCCTCT**\|**AAAACCAAAACAAGGCCAAGGTTGAGCTCTAGTTGGCAGAATGCTCATCAGGTAGTTCTTGGAGGCTTTGAATCTGGAATCCCCTAGCCCCAGCCTTCTAGGAGCTAGATTACAAGCAAGTGCTGTTTCTATTAAATACCATGTTTTGGGTTTGGGGAGATGGGCCAGTGGTCAAGAGTGCTTGCTTCTCCTTCA |
| ∆OS_SRR107+111/+ (OS intact)  chr3:34758043-34758115  the intact OS motif is marked in bold with an underline | A2 | N | CTTCTGGGTGGTGAACCTTGGCA**\|**CATAATGGGGCT**AAATAAATAACAATG\|**GGACTATGCTAACCTTCCTGGGTAACAGCCGGGAGGGAGGTGTCATT |
|  | E2 | N | CTTCTGGGTG**\|**GGGGC**TAATAAATAACAATG**ACAGTACTTGCCCTTAGCAGAGTCCTGGGACTATGCTAAACAACTTCCTGGGTAACAGCCGGGAGGGAGGTGTC |
|  | H8 | N | CTTCTGGGTGGTGAA**\|**CCTTGGCA**\|**TATTATTTTTAGC**\|**TAATGGGGC**TAA\|TAAATAACAATG**A**\|**ACTTGCC**\|**TTAC**\|**AGTCCTGGGACTATGCTAA**\|**CAACTTCCTGGG**\|**AC**\|**GCCGGGAGGGAGGTGTCATT |
| ∆OS_SRR107+111/+  chr3:34758043-34758115 | B1 | N | CTTCTGGGTGGTGAACCTTGGC\|TATGCTAAACAACTTCCTGGGTAACAGCCGGGAGGGAGGTGTCATT |
|  | C2 | N | CTTCTGGGTGGTGAACCTTGGCA**\|**CCTGGGACTATGCTAAACAACTTCCTGGGTAACAGCCGGGAGGGAGGTGTCATT |
|  | F5 | N | CTTCTGGGTGGTGAACCTTGG**\|**CAGAGTCCTGGGACTATGCTAAACAACTTCCTGGGTAACAGCCGGGAGGGAGGTGTCATT |
| ∆SRR107+ K(2)_111/+  chr3:34761207-34761274 | B1 | N | AGCCAGAGATAACCTGGTGGTTGAAGGCAGCCTTC**\|**CCAGGGTGCCAACTTTGAAGGGCCACAGTAAAGATTAAATTGTATGTCCCACCTTTATAGCACTCAGGGGGCTGAGGCAGGAGCATCAGGAATCCGAGGCCTTAGCTACGAAACAGGTTCGAGACCAGCTGCAGTTACAAGAAACCCTCTCTCAATTTCAATGTCCTGTACCCCACCAA |
|  | B11 | N | TCACCTTGAGCCAGAGATAACCTGGT**\|**TAGG**\|**TCATCTCATCTTCTAAACCATCCCAGGGTGCCAACTTTGAAGGGCCACAGTAAAGATTAAATTGTATGTCCCACCTTTATAGCACTCAGGGGGCTGAGGCAGGAGCATCAGGAATCCGAGGCCTTAGCTACGAAACAGGTTCGAGACCAGCTGCAGTTACAAGAAACCCTCTCTCAATTTCAATGTCCTGTACCCCACCAA |
|  | D1 | N | GTCACCTTGAGCCAGAGATAACCTGGTGGTTGAAGGCAGCCTTC**\|**ATCTCATCTTCTAAACCATCCCAGGGTGCCAACTTTGAAGGGCCACAGTAAAGATTAAATTGTATGTCCCACCTTTATAGCACTCAGGGGGCTGAGGCAGGAGCATCAGGAATCCGAGGCCTTAGCTACGAAACAGGTTCGAGACCAGCTGCAGTTACAAGAAACCCTCTCTCAATTTCAATGTCCTGTACCCCACCAA |
|  | G2 | N | CCTTGAGCCAGAGATAACCTG**\|**CCTTTCATCTCATCTTCTAAACCATCCCAGGGTGCCAACTTTGAAGGGCCACAGTAAAGATTAAATTGTATGTCCCACCTTTATAGCACTCAGGGGGCTGAGGCAGGAGCATCAGGAATCCGAGGCCTTAGCTACGAAACAGGTTCGAGACCAGCTGCAGTTACAAGAAACCCTCTCTCAATTTCAATGTCCTGTACCCCACCAA |
|  | H9 | N | GAGGGTTCTTGTACTGCAGCTGGTCTCGAACCTGTTTCGTAGCTAAGGCCTCGGATTCCTGATGCTCCTGCCTCAGCCCCCTGAGTGCTATAAAGGTGGGACATACAATTTAATCTTTACTGTGGCCCTTCAAAGTTGGCACCCTGGGATGGTTTAGAAGATGAGATGAAA**\|**CCTCGTTAATAGAAGAATTTAAGAATGACTCAAATGGAAGGTGGAGGACAATTAGGGTTTAAAAAAAGAACCTGGGATGGGCCAGTTGTAAACCCCCTGGAGCTGCCTAGAGGAAGGAGCTGGAGGAGAGCTTAGAAAACAAAGGGGGAGGTCATGGAAACAGACGGGGAGGTCAGACA |
| ∆SRR85-95/+  chr3:34733021-34748441 | E2 | N | CTGCATGGAAGTTCCTAGACCAGTGTCTGGTGCTCTGGAGTGAGTGATGTCACTGGGTTCTGGATATCAGGTGCAGCCA**\|**TGCGTGTCATACTGTTTTAAGATCAGAAATGCTAAAGGTTCAGTCAATTTTCATGGTTCTACTTTGACACTCTCCCGCAGAACTTATGGTCTGTTTAAAAATAGAAAACGCAGCCATCTGGCTATTTGATGGGATTCTATTTTTGTTTCTCTTTGCGTTCGCAAAGTGTGTTGGGTCTGAAATTTTCCGTGTTCTGCCCTATATGTAATTGTGTGTATATACACACATACTTTCTCATTTAAATCTCCATACACTTCCTCATCTAAATCTTGCTGTTATCAGTCTGTGTTGTTTGTGGTCCACGGCAGTGTTTGGTCGGGATGTCAACCTTGCTTAGTTCATCCACTAGTCACGTCTGCACTGAATTCCTACTCTAAATTCTTACCAAA |
|  | F6 | N | TGCATGGAGTTCTAGACCAGTGTCTGGTGCTCTGGAGTGAGTGATGTCACTGGGTTCTGGATATCAGGTGCAGCCATGG**\|**CACTGCGTGTCATACTGTTTTAAGATCAGAAATGCTAAAGGTTCAGTCAATTTTCATGGTTCTACTTTGACACTCTCCCGCAGAACTTATGGTCTGTTTAAAAATAGAAAACGCAGCCATCTGGCTATTTGATGGGATTCTATTTTTGTTTCTCTTTGCGTTCGCAAAGTGTGTTGGGTCTGAAATTTTCCGTGTTCTGCCCTATATGTAATTGTGTGTATATACACACATACTTTCTCATTTAAATCTCCATACACTTCCTCATCTAAATCTTGCTGTTATCAGTCTGTGTTGTTTGTGGTCCACGGCAGTGTTTGGTCGGGATGTCAACCTTGCTTAGTTCATCCACTAGTCACGTCTGCACTGAATTCCTACTCTAAATTCTTACCAAAGGTCCCC |
|  | G4 | N | TGTCAAGTGGACTGCATGGAGTTCCTAGACCAGTGTCTGGTGCTCTGGAGTGAGTGATGTCACTGGGTTCTGGATATCAGGTGCAGCCATGGG**\|**CACTGCGTGTCATACTGTTTTAAGATCAGAAATGCTAAAGGTTCAGTCAATTTTCATGGTTCTACTTTGACACTCTCCCGCAGAACTTATGGTCTGTTTAAAAATAGAAAACGCAGCCATCTGGCTATTTGATGGGATTCTATTTTTGTTTCTCTTTGCGTTCGCAAAGTGTGTTGGGTCTGAAATTTTCCGTGTTCTGCCCTATATGTAATTGTGTGTATATACACACATACTTTCTCATTTAAATCTCCATACACTTCCTCATCTAAATCTTGCTGTTATCAGTCTGTGTTGTTTGTGGTCCACGGCAGTGTTTGGTCGGGATGTCAACCTTGCTTAGTTCATCCACTAGTCACGTCTGCACTGAATTCCTACTCTAAATTCTTACCA |
| ∆SRR104-dCTCF/+  chr3:34755000-34774122 | B6 | Y | GTAGTGACTGCAGCAGACTTGGGAAGATACTTTACCATCCCACAGCTGAGAGCCACTGAGACCGAGGTTTAGAATTTCATCCTCAAGCCAAGATACTAAACATATCAATGAATGCGGATGCCTTGCTATGCCCAGAATTCCCTCTCCGTCTCCAAGCCTTACGGGAACGCCATATGCCAGGGGTTCCTGGCAGCAGGAAACCAAGAGACTAACAGAATAAATTACTTTACATTAGACACGTGCTGTTGACCTGCTCGAGGTATGAAG**\|**TGGTTAGAGCACTTACTGCTTTTCCAGGGGACCTGGGATGGCTCCTCCCCACCCACATGGTGGTTCAGAGCTGACTGTAACTCTAGCCAGGGGATGTGTTGTCCTCTTCTGGCCTCTGCCTACAGCAAGTGCCCACATATATGTGCAGGTATGTATATGATGGCATATGATCCCAGCAATC |
|  | B8 | N | TCCAAGCCAAGGCTCAGCGACTCTGAGTCCCAACATCACTGTAGTGACTGCAGCAGACTTGGGAAGATACTTTACCATCCCACAGCTGAGAGCCACTGAGACCGAGGTTTAGAATTTCATCCTCAAGCCAAGATACTAAACATATCAATGAATGCGGATGCCTTGCTATGCCCAGAATTCCCTCTCCGTCTCCAAGCCTTACGGGAACGCCATATGCCAGGGGTTCCTGGCAGCAGGAAACCAAGAGACTAACAGAATAAATTACTTTACATTAGACACGTGCTGTTGACCTGCTCGAGGTATGAAGAATATTAACACCGTCCCCG**\|**GTAGCTCAGTGGTTAGAGCACTTACTGCTTTTCCAGGGGACCTGGGATGGCTCCTCCCCACCCACATGGTGGTTCAGAGCTGACTGTAACTCTAGCCAGGGGATGTGTTGTCCTCTTCTGGCCTCTGCCTACAGCAAGTGCCCACATATATGTGCAGGTATGTATATGATGGCATATGATCCCAGCAATC |
|  | E6 | N | CCAAGGCTCAGCGACTCTGAGTCCCAACATCACTGTAGTGACTGCAGCAGACTTGGGAAGATACTTTACCATCCCACAGCTGAGAGCCACTGAGACCGAGGTTTAGAATTTCATCCTCAAGCCAAGATACTAAACATATCAATGAATGCGGATGCCTTGCTATGCCCAGAATTCCCTCTCCGTCTCCAAGCCTTACGGGAACGCCATATGCCAGGGGTTCCTGGCAGCAGGAAACCAAGAGACTAACAGAATAAATTACTTTACATTAGACACGTGCTGTTGACCTGCTCGAGGTATGAAGAATATTAACACCGTCCCCGAACGG**\|**TAGCTCAGTGGTTAGAGCACTTACTGCTTTTCCAGGGGACCTGGGATGGCTCCTCCCCACCCACATGGTGGTTCAGAGCTGACTGTAACTCTAGCCAGGGGATGTGTTGTCCTCTTCTGGCCTCTGCCTACAGCAAGTGCCCACATATATGTGCAGGTATGTATATGATGGCATATGATCCCAGCAA |
| ∆SRR85-95+SCR-dCTCF/+  chr3:34733021-34748441  +  chr3:34755000-34774122 | B7 | N | CAAGCCAAGGGCTCAGCGACTCTGAGTCCCAACATCACTGTAGTGACTGCAGCAGACTTGGGAAGATACTTTACCATCCCACAGCTGAGAGCCACTGAGACCGAGGTTTAGAATTTCATCCTCAAGCCAAGATACTAAACATATCAATGAATGCGGATGCCTTGCTATGCCCAGAATTCCCTCTCCGTCTCCAAGCCTTACGGGAACGCCATATGCCAGGGGTTCCTGGCAGCAGGAAACCAAGAGACTAACAGAATAAATTACTTTACATTAGACACGTGCTGTTGACCTGCTCGAGGTATGAAGAATATTAACACCGTCCCCGAACGG**\|**TAGCTCAGTGGTTAGAGCACTTACTGCTTTTCCAGGGGACCTGGGATGGCTCCTCCCCACCCACATGGTGGTTCAGAGCTGACTGTAACTCTAGCCAGGGGATGTGTTGTCCTCTTCTGGCCTCTGCCTACAGCAAGTGCCCACATATATGTGCAGGTATGTATATGATGGCATATG |
|  | C11 | Y | GGAAGATACTTTACCATCCCACAGCTGAGAGCCACTGAGACCGAGGTTTAGAATTTCATCCTCAAGCCAAGATACTAAACATATCAATGAATGCGGATGCCTTGCTATGCCCAGAATTCCCTCTCCGTCTCCAAGCCTTACGGGAACGCCATATGCCAGGGGTTCCTGGCAGCAGGAAACCAAGAGACTAACAGAATAAATTACTTTACATTAGACACGTGCTGTTGACCTGCTCGAGGTATGAAGAATATTAACACCGTCCCCGAACGG**\|**TAGCTCAGTGGTTAGAGCACTTACTGCTTTTCCAGGGGACCTGGGATGGCTCCTCCCCACCCACATGGTGGTTCAGAGCTGACTGTAACTCTAGCCAGGGGATGTGTTGTCCTCTTCTGGCCTCTGCCTACAGCAAGTGCCCACATATATGTGCAGGTATGTATATGATGGCATATGATC |
|  | D2 | N | GTAGTGACTGCAGCAGACTTGGGAAGATACTTTACCATCCCACAGCTGAGAGCCACTGAGACCGAGGTTTAGAATTTCATCCTCAAGCCAAGATACTAAACATATCAATGAATGCGGATGCCTTGCTATGCCCAGAATTCCCTCTCCGTCTCCAAGCCTTACGGGAACGCCATATGCCAGGGGTTCCTGGCAGCAGGAAACCAAGAGACTAACAGAATAAATTACTTTACATTAGACACGTGCTGTTGACCTGCTCGAGGTATGAAGAAT**\|**AACGGTAGCTCAGTGGTTAGAGCACTTACTGCTTTTCCAGGGGACCTGGGATGGCTCCTCCCCACCCACATGGTGGTTCAGAGCTGACTGTAACTCTAGCCAGGGGATGTGTTGTCCTCTTCTGGCCTCTGCCTACAGCAAGTGCCCACATATATGTGCAGGTATGTATATGATGGCATATGAT |
| ∆SRR85-95+107-dCTCF/+  chr3:34733021-34748441  +  chr3:34757641-34774122 | A12 | Y | TAAAGTTTAAACGTACATTTTTTTTTCATTTTTTATTAGGTATTTAGCTCATTTACATTTCCAATGCTATACCAAAAGTCCCCCATACCCACCCAACG**\|**TCGGGGACGGTGTTAATATTCTTCATACCTCGAGCAGGTCAACAGCACGTGTCTAATGTAAAGTAATTTATTCTGTTAGTCTCTTGGTTTCCTGCTGCCAGGAACCCCTGGCATATGGCGTTCCCGTAAGGCTTGGAGACGGAGAGGGAATTCTGGGCATAGCAAGGCATCCGCATTCATTGATATGTTTAGTATCTTGGCTTGAGGATGAAATTCTAAACCTCGGTCTCAGTGGCTCTCAGCTGTGGGATGGTAAAGTATCTTCCCAAGTCTGCTGCAGTCACTACAGTGATGTTGGGACTCAGAGTCGCTGAGCCTTGGCTTGGAGACCTGATAAGGGCTTGTAAGAGTAGTACCTCAGTCTCCCTAAGGCCTGCCTGGAGTTCTGCACTGCAACTGTGTCCGAGGAGTCCTCCCTTAA |
|  | C3 | N | CAATTCATCATCAAGACATCATGATTTTGAGTTTAAACGTACATTTTTTTTTCATTTTTTATTAGGTATTTAGCTCATTTACATTTCCAATGCTATACCAAAAGTCCCCCATACCCACCCAACG**\|**TCGGGGACGGTGTTAATATTCTTCATACCTCGAGCAGGTCAACAGCACGTGTCTAATGTAAAGTAATTTATTCTGTTAGTCTCTTGGTTTCCTGCTGCCAGGAACCCCTGGCATATGGCGTTCCCGTAAGGCTTGGAGACGGAGAGGGAATTCTGGGCATAGCAAGGCATCCGCATTCATTGATATGTTTAGTATCTTGGCTTGAGGATGAAATTCTAAACCTCGGTCTCAGTGGCTCTCAGCTGTGGGATGGTAAAGTATCTTCCCAAGTCTGCTGCAGTCACTACAGTGATGTTGGGACTCAGAGTCGCTGAGCCTTGGCTTGGAGACCTGATAAGGGCTTGTAAGAGTAGTACCTCAGTCTCCCTAAGGCCTGCCTGGAGTTCTGCAAAAAAACTGTGTCAAAAGAAAAAC |
|  | D12 | N | AATTCATCATCAAGACATCATGATTTTGAGTTTAAACGTACATTTTTTTTTCATTTTTTATTAGGTATTTAGCTCATTTACATTTCCAATGCTATACCAAAAGTCCCCCATACCCACCCAACG**\|**TCGGGGACGGTGTTAATATTCTTCATACCTCGAGCAGGTCAACAGCACGTGTCTAATGTAAAGTAATTTATTCTGTTAGTCTCTTGGTTTCCTGCTGCCAGGAACCCCTGGCATATGGCGTTCCCGTAAGGCTTGGAGACGGAGAGGGAATTCTGGGCATAGCAAGGCATCCGCATTCATTGATATGTTTAGTATCTTGGCTTGAGGATGAAATTCTAAACCTCGGTCTCAGTGGCTCTCAGCTGTGGGATGGTAAAGTATCTTCCCAAGTCTGCTGCAGTCACTACAGTGATGTTGGGACTCAGAGTCGCTGAGCCTTGGCTTGGAGACCTGATAAGGGCTTGTAAGAGTAGTACCTCAGTCTCCCTAAGGCCTGCCTGGAGTTCTGCACAGCAACTGTGTCCAAGGA |
|  | H1 | N | TTCTTCATCAAGACATCATGATTTTGAGTTTAAACGTACATTTTTTTTTCATTTTTTATTAGGTATTTAGCTCATTTACATTTCCAATGCTATACCAAAAGTCCCCCATACCCACCCAACG**\|**TCGGGGACGGTGTTAATATTCTTCATACCTCGAGCAGGTCAACAGCACGTGTCTAATGTAAAGTAATTTATTCTGTTAGTCTCTTGGTTTCCTGCTGCCAGGAACCCCTGGCATATGGCGTTCCCGTAAGGCTTGGAGACGGAGAGGGAATTCTGGGCATAGCAAGGCATCCGCATTCATTGATATGTTTAGTATCTTGGCTTGAGGATGAAATTCTAAACCTCGGTCTCAGTGGCTCTCAGCTGTGGGATGGTAAAGTATCTTCCCAAGTCTGCTGCAGTCACTACAGTGATGTTGGGACTCAGAGTCGCTGAGCCTTGGCTTGGAGACCTGATAAGGGCTTGTAAGAGTAGTACCTCAGTCTCCCTAAGGCCTGCCTGGAGTTCTGCACTGCAACTGTG |

**Supplementary Table S4: Guide sequences for insertion lines**

| Name | Target site | Sequence |
| --- | --- | --- |
| *Sox2* | 3’ coding sequence of *Sox2* | CCCCTGTCGCACATGTGA |
| MH5’ | 5’ of P2A-Venus cassette | TTCCTCCCATGTGCGCCC |
| MH3’ | 3’ of P2A-Venus cassette | CAAGTAATGAGGGCTCCC |
| Insertion | Intervening region between *Sox2* and SCR | GTTCAAAAACTAGAAACA |

**Supplementary Table S5: qPCR primers for gene expression analysis (SNPs indicated as lowercase)**

| mRNA | Allele | Forward Sequence | Reverse Sequence |
| --- | --- | --- | --- |
| Sox2 | 129 | GGACTTCTTTTTGGGGGACT | CGCCTAACGTACCACTAGAACTT**t** |
| Sox2 | CAST | GGACTTCTTTTTGGGGGACT | CGCCTAACGTACCACTAGAACTT**a** |
| Sdha | n/a | ACTGGGATGGGCTCCTTAGT | GCCCTGAGAAAGATCACGTC |
| Gapdh | n/a | GCACCAGCATCCCTAGACC | CTTCTTGTGCAGTGCCAGGTG |

**Supplemental Table S6: 4C primers**

Sequences of 4C primers. Blue denotes Illumina adapter sequence for high-throughput sequencing. Red denotes position of 6-nucleotide barcodes, used to multiplex 4C samples for sequencing.

| **Name** | **Sequence** |
| --- | --- |
| Near-SCR DpnII | 5’-AATGATACGGCGACCACCGAGATCTACACTCTTTCCCTACACGACGCTCTTCCGATCTNNNNNNGCAAGAGCCAGGTGTGGCTC-3’ |
| Near-SCR Csp6I | 5’- CAAGCAGAAGACGGCATACGAGCTCTTCCGATCTCCTGGTGCTTTGCCCAGCAC-3’ |
| SCR DpnII | 5’- AATGATACGGCGACCACCGAGATCTACACTCTTTCCCTACACGACGCTCTTCCGATCTNNNNNNGGGGAGGTCAGACACCTGATC-3’ |
| SCR Csp6I | 5’- CAAGCAGAAGACGGCATACGAGCTCTTCCGATCTTTCCGGTAGGGGTGGAGC-3’ |
| hSOX9 DpnII | 5’- AATGATACGGCGACCACCGAGATCTACACTCTTTCCCTACACGACGCTCTTCCGATCTNNNNNNAGGACATTGATTTGGATC-3’ |
| hSOX9 Csp6I | 5’-CAAGCAGAAGACGGCATACGAGCTCTTCCGATCTCGTAGTGTGGACCTATTT-3’ |
| *Sox2* NlaIII | 5’- AATGATACGGCGACCACCGAGATCTACACTCTTTCCCTACACGACGCTCTTCCGATCTNNNNNNAAATACAAAAACTATAGAAA |
| *Sox2* Csp6I | 5’-CAAGCAGAAGACGGCATACGAGCTCTTCCGATCTTCCAGAATTAGAAAGCCTTT |

**Supplemental Table S7: ChIP-qPCR primers (SNPs indicated as lowercase).**

| **Target Site** | **Allele** | **Forward Sequence** | **Reverse Sequence** |
| --- | --- | --- | --- |
| OS_SRR107 | 129 | ATTTCCTTGTGTCCAGCTATAAa | TTTGCAGCGACTTCTCCTG |
| OS_SRR107 | Cast | GCTTCTCACAAAACAAAATGACTAt | AGGTTTTCGTTGGTGGAGAC |
| hSOX9 | n/a | agaaattctcccggaaggac | cttcggaataggaacttcgc |
| hSOX9 -2 kb | n/a | acgaccagaatccccaaag | gcatcctcatctcacagaac |
| SRR109 | n/a | atctggcttactgaggctc | gcctggtgaagtatctgacg |
| SRR109 -2 kb | n/a | ccaaaaccaacaaccaaacc | gtcttgctgaagagaactaacc |
